# Putatively Adaptive and Genome‐Wide Genetic Variation Often Rank Populations as Similarly Distinctive

**DOI:** 10.1111/mec.70483

**Published:** 2026-08-04

**Authors:** Avneet K. Chhina, Philippe Fernandez‐Fournier, Jayme M. M. Lewthwaite, Tom R. Booker, Arne Mooers

**Affiliations:** ^1^ Department of Biological Sciences Simon Fraser University Burnaby British Columbia Canada; ^2^ Department of Biology Carleton University Ottawa Ontario Canada; ^3^ Department of Entomology Cornell University Ithaca New York USA; ^4^ Department of Forest and Conservation Sciences University of British Columbia Vancouver British Columbia Canada

**Keywords:** adaptive genetic variation, adaptive potential, comparative study, conservation genetics, genome‐wide genetic variation, population distinctiveness, population prioritization

## Abstract

Identifying which populations within species to prioritize to maintain genetic diversity is challenging. Due to its direct connection to current patterns of adaptation and presumed connection to adaptive capacity, some researchers advocate prioritizing adaptive variation. Other researchers advocate prioritizing populations using genome‐wide variation, citing the many limitations in identifying adaptive genetic variation, an unknown future, and verbal theory that suggests patterns of easy‐to‐gather genome‐wide genetic variation and adaptive genetic variation may often be congruent. However, evidence for such congruence is mixed. We gathered genome‐wide and putatively adaptive genetic data (using single nucleotide polymorphisms; SNPs) across 34 published outlier and association studies of plants and animals to test congruence. We asked directly whether putatively adaptive subsets of genome‐wide SNPs identify similar sets of distinctive populations (measured using the Shapley value of distinctiveness) as do sets of genome‐wide SNPs. We find that genome‐wide and putatively adaptive SNPs generally agree on population prioritizations, with the level of agreement predicted by the proportion of putatively adaptive SNPs in the dataset. Unexpectedly, the prioritization agreement between putatively adaptive SNPs and genome‐wide SNPs is stronger than that between random sets of SNPs and genome‐wide SNPs. Overall, using genome‐wide genetic variation may be a generally sound and cost‐effective strategy for prioritizing populations in order to safeguard the species‐level genetic variation needed for ongoing adaptation.

## 1 Introduction

The United Nations Post‐2020 Global Biodiversity Framework includes as an aim to maintain ‘at least 90% of genetic diversity within all species’ (Convention on Biological Diversity (CBD) 2021); however, recent studies suggest we may already be falling short (Exposito‐Alonso et al. 2022; Frankham 2022). This increases the urgency with which we must tackle the issue of conserving intraspecific genetic variation, both for conservation (Hoban et al. 2020; Laikre et al. 2020; Exposito‐Alonso et al. 2022) and in the context of resource management (Hoban et al. 2020; Hoban et al. 2021). Due to limited funds, prioritizing particular populations so as to maintain distinct pools of genetic variation may be an important component of conservation and resource management (Wilson 1988; Millar and Libby 1991; Vane‐Wright et al. 1991; Waples 1991; Crozier 1992; Faith 1992; Lesica and Allendorf 1995; Bottrill et al. 2008; Taylor et al. 2011; Coleman et al. 2013; McMahon et al. 2014; Volkmann et al. 2014; Mable 2019; Hoban et al. 2020; Allendorf et al. 2022). For instance, these genetically distinct populations may possess variation that would facilitate adaptation to future conditions at the species level (Coates et al. 2018; Dıaz et al. 2020; Hoban et al. 2020).

However, identifying adaptive genetic variation is difficult (recently reviewed in Chhina et al. 2024; see also Slavov et al. 2025) and may not be necessary. Conservation practitioners are generally constrained due to limited resources (in budget and in time) in what they can measure genetically. Studies aiming to identify adaptive variants have typically relied on two broad classes of methods. First, F_ST_‐outlier approaches detect loci exhibiting higher‐than‐expected population differentiation under the assumption that such loci may be under divergent selection. Second, genotype–environment association (GEA) methods identify loci whose allele frequencies are statistically associated with environmental gradients, suggesting local adaptation to divergent environments. These approaches differ in their assumptions and limitations: F_ST_‐based methods do not make explicit assumptions about the drivers of adaptation, while GEA methods may be confounded by covariation between population structure and the environment. Both classes of methods may be confounded by non‐equilibrium demographic histories (Lotterhos and Whitlock 2014). Recent studies highlight the limitations of these approaches in identifying adaptation to novel climatic environments (Lind and Lotterhos 2024; Li et al. 2025; Slavov et al. 2025): current methods can be biased, challenging, expensive, and time‐consuming (Chung et al. 2021; Harrisson et al. 2014). Several studies have noted the congruence in genetic patterns when using putatively adaptive single nucleotide polymorphisms (SNPs) and easier‐to‐gather genome‐wide or putatively neutral SNPs (see, e.g., Li et al. 2025, Mathur et al. 2023, Moore et al. 2014, Oh et al. 2019, Xuereb et al. 2022). Congruence between the two types of markers can be affected by many factors. If the number of SNPs surveyed is low, statistical power is low (Morin et al. 2009; Smouse 2010; Landguth et al. 2012), inflating or deflating the correlation by chance. If identified adaptive SNPs are few, they may not capture the breadth of selection across the genome or across environments. And, finally, methods for detecting selection often have high false positive/negative rates (François et al. 2016). Given these issues, the debate over which set of markers is more important is rich (Ryder 1986; Hard 1995; Moore et al. 2014; Bertin et al. 2020; DeWoody et al. 2021; Fitzpatrick et al. 2021; García‐Dorado and Caballero 2021; Kardos et al. 2021; Teixeira and Huber 2021) and has implications for strategies such as assisted gene flow (Aitken and Whitlock 2013).

Fernandez‐Fournier et al. (2021) explicitly asked whether genome‐wide genetic variation and the subset of that identified as adaptive led to similar measures of population distinctiveness. They considered two published SNP datasets—for yellow warbler (Bay et al. 2018) and for lodgepole pine (Mahony et al. 2020)—and found that genome‐wide and putatively adaptive genetic distinctiveness produced similar population rankings for both species. Here we test the generality of this pattern by extending Fernandez‐Fournier et al. (2021) to a larger set of species with varying natural histories. With this larger dataset we ask the following questions: (1) Do genome‐wide SNPs and putatively adaptive SNPs provide similar population prioritizations? (2) Given that putatively adaptive SNPs are a subset of genome‐wide SNPs, do they behave like equal‐sized samples of random SNPs? And (3) do simple study‐level characteristics impact the prioritization agreement between the two sets of markers?

## 2 Methods

We focused on genetic SNP data, and measured genetic distinctiveness in two ways: genome‐wide and putatively adaptive. Genome‐wide genetic distinctiveness was computed from all SNPs, regardless of context, potentially including neutral, deleterious, and adaptive genetic variation. Adaptive genetic distinctiveness was computed from a subset of this genome‐wide variation that has been associated with natural selection in the past (Holderegger et al. 2006; Schoville et al. 2012). Authors define ‘putatively adaptive’ in different ways depending on the type of analysis, study system, and study specifics, so this category may include very different definitions. Two common methods to identify putatively adaptive genetic variants are outlier‐type analyses that identify variants exhibiting higher differentiation among populations than the background *F*_*ST*_ expected under neutrality in genome scans (Lewontin and Krakauer 1973; Beaumont and Nichols 1996; Lotterhos and Whitlock 2014; Ahrens et al. 2018), and association‐type analyses that detect genetic variants associated with environmental or phenotypic variation across geographical space (see, e.g., Hoban et al. 2016). Each method has strengths and weaknesses (Savolainen et al. 2013; Chhina et al. 2024).

Tools to identify *F*_*ST*_ outliers in our studies include BayeScan (Foll and Gaggiotti 2008), FDIST2 (Beaumont and Nichols 1996), BayesFst/Lositan (Beaumont and Nichols 1996; Antao et al. 2008; Excoffier et al. 2009), Arlequin (Excoffier et al. 2005; Excoffier et al. 2009), OutFlank (Whitlock and Lotterhos 2015) and FLK (Bonhomme et al. 2010). Association‐type approaches included LFMM (Frichot et al. 2013; Frichot and François 2015), RDA (Legendre and Gallagher 2001), Bayenv2 (Günther and Coop 2013), PCA‐adapt (Duforet‐Frebourg et al. 2014), BayeScEnv (de Villemereuil and Gaggiotti 2015), Matsam/Sam (Joost et al. 2007; Joost et al. 2008), Clinality (Berry and Kreitman 1993), Bayesian sparse linear mixed models (Zhou and Stephens 2012; Zhou et al. 2013), Sambada (Stucki et al. 2016), linear regression‐based MLMA in GCTA (Yang et al. 2011), MLM (Yu et al. 2005), Unified mixed models (Balding 2006; Yu et al. 2005), and GWAS (Bradbury et al. 2007). Different software tools and approaches vary in sensitivity and underlying assumptions, including specific demographic models, equilibrium conditions, and other biological and statistical considerations. The methodological differences among these different software tools and approaches will impact the identification of adaptive SNPs and so concordance patterns. Whereas some methods are more conservative (introducing false negatives and thereby reducing concordance if true adaptive loci are missed), others are prone to more false positives (inflating concordance if non‐adaptive loci are misclassified). Detailed comparisons of underlying assumption and the pros and cons of some detection approaches are outlined in Lotterhos and Whitlock (2014), Excoffier et al. (2009), and Hoban et al. (2016). Given the range of approaches and our limited sample size it is challenging to compare the various methodological differences used to identify putatively adaptive SNPs; instead, we examined whether broad classes of methods used to identify adaptive SNPs (*F*_*ST*_‐outlier, GEA, GPA) impact correlations between prioritization rankings (see section 2.3.1 Study characteristics impacting rank correlation).

### 2.1 Study Selection and Data Filtering

We collated SNP data from published studies that employed outlier‐type (*F*_*ST*_), genotype‐environment association (GEA) and/or genotype–phenotype association (GPA) methods to identify putatively adaptive genetic variation in wild species. Studies were sourced from previous meta‐analyses (Ahrens et al. 2018; Lind et al. 2018; Waldvogel et al. 2020; Exposito‐Alonso et al. 2022) supplemented with results from database keyword searches. We only considered studies that contained accessible genetic data that included lists of SNPs identified as putatively adaptive for multiple individuals with geographic coordinates. We then filtered studies that had a total of at least 250 SNPs, with no fewer than 10 raw putatively adaptive SNPs and at least 50 individuals with at least 4 individuals in each of 4 populations. When multiple studies for a species existed, we chose the study with the broader geographic sampling. Studies that sampled a very limited section of a species' geographic range (roughly less than 5% of the natural geographic range, from a visual inspection) were not included. Supporting Information contains relevant information on included studies.

We converted SNP files from different file formats into a common format where each SNP genotype was coded as the number of minor alleles present (i.e., 0,1,2, with all minor alleles pooled). To filter out SNPs and samples with missing data, we followed Fernandez‐Fournier et al. (2021) and used a threshold of 10% for missing alleles per SNP and for missing SNPs per sample (Troyanskaya et al. 2001; Stacklies et al. 2007; Stacklies and Redestig 2022), using the package pcaMethods and function ‘svdImpute’ (Troyanskaya et al. 2001). If the number of individuals per population after accounting for the 10% missingness rate (i.e., samples and SNPs) fell below four, we removed that population.

### 2.2 Population Distinctiveness Rankings

**FIGURE 1:**
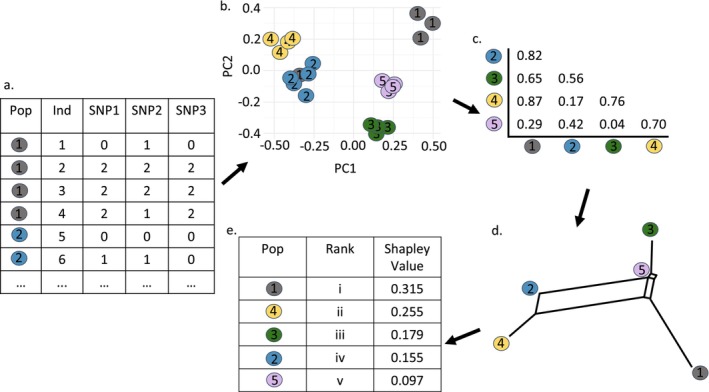
Overview of the analytical approach taken for data organization and data analysis. For each study and set of genetic variants (genome‐wide, putatively adaptive, and the 1000 random subsets of genome‐wide SNPs), we obtained a SNP dataset of all individuals within populations (a), performed a principal components analysis (b), and measured pairwise genetic differentiation among populations (c). Split information from NeighborNet (d) was used to calculate a distinctiveness score (Shapley value) and to rank populations (e).

We analyzed each study following the methods used by Fernandez‐Fournier et al. (2021), illustrated in Figure 1. We first performed a principal components analysis (PCA) (Pearson 1901; Hotelling 1933) on a given set of SNPs for each study. We identified and retained significant principal components (PCs) using the Tracy‐Widom test (Tracy and Widom 1994) with a critical point of 0.9793 (see Patterson et al. 2006; Paschou et al. 2007; Ma and Amos 2012), but always kept at least one PC. Using population assignments from the original publications, we then computed the pairwise Euclidean distances among population centroids. We did this separately for the genome‐wide, the putatively adaptive, and the random subsets of genome‐wide SNPs datasets (see below). One to two outlier individuals from each of three studies (Chávez‐Galarza et al. 2013; Benestan et al. 2016; Mosca et al. 2016) were removed before calculating these pairwise distances, based on them being > 4 standard deviations from the mean in overall PC space.

Using the inferred pairwise genetic distances among populations, we built a circular split network (Huson and Bryant 2006) for each set of SNPs within each species. A circular split network depicts relationships among populations that accommodate both tree‐like and non‐tree‐like patterns of similarity (Bryant and Moulton 2002; Bryant and Moulton 2004; Volkmann et al. 2014). We used NeighborNet (Bryant and Moulton 2002) as implemented in the R phangorn package (Schliep 2011; Schliep et al. 2017), and for confirmation, the SplitsTree software (Huson and Bryant 2024). Populations that are more distinctive are on longer branches (contribute to larger splits) in the network.

Finally, we calculated each population's Shapley value (Haake et al. 2008; Fernandez‐Fournier et al. 2021; Volkmann et al. 2014) as a measure of its genetic distinctiveness. In the context of our study, the Shapley value of a population is its expected contribution to future circular split networks made up of all possible subsets of the other populations (Volkmann et al. 2014). Populations with higher Shapley values are more distinctive, and contribute more, on average, to the total genetic diversity as represented by the circular split network. We used these scores to create distinctiveness rankings.

### 2.3 Statistical Analyses

We tested whether similar populations were prioritized based on genome‐wide vs. putatively adaptive genetic distinctiveness across studies by calculating the correlation between rankings using each set of SNPs (Figure 1). We first calculated the pairwise Spearman rank correlation (*r*_*s*_) of genome‐wide vs. putatively adaptive SNP‐based distinctiveness rankings, which we refer to as the observed correlation (along with the 90% bootstrapped confidence interval from the RVAideMemoire R package; Hervé 2020).

Putatively adaptive SNPs are a subset of the genome‐wide SNPs. To test whether this subset of the total variation behaves like equal‐sized samples of randomly‐selected SNPs, we constructed 1000 random samples matching the number of the putatively adaptive SNPs identified for each study. We refer to this as the expected correlation, which we can compare with the observed correlation. The observed correlation minus expected correlation indicates the predictive power of the putatively adaptive SNPs: negative values indicate that putatively adaptive SNPs show a weaker correlation with genome‐wide SNPs compared to random sets of SNPs, values close to zero indicate that putatively adaptive SNPs and random sets of SNPs correlate equally with genome‐wide SNPs, and positive values indicate that putatively adaptive SNPs show a stronger correlation with genome‐wide SNPs than random sets of SNPs.

We performed a two‐tailed paired *t*‐test to test for a difference between observed and expected Spearman correlation coefficients.

#### 2.3.1 Study Characteristics Impacting Rank Correlation

We scored studies for the genomic analysis method used (*F*_*ST*_, GEA, GPA) and the specific statistical packages used and analysis type, the latter to capture whether the authors attempted to account for neutral population structure or demography or whether they applied other approaches to reduce the false‐positive rate (see Supporting Information File B). Including these, we tested the individual effects of six characteristics that might impact the observed correlation between genome‐wide and putatively adaptive SNP‐based conservation prioritizations.

First, we included population structure, as estimated via study‐wide *F*_*ST*_. We predicted that species with more population structure (higher *F*_*ST*_) would show stronger prioritization agreement, under the assumption that the processes producing structure would affect neutral and adaptive variation in concert. We used guidelines found at (Schluter n.d.) to calculate study‐wide *F*_*ST*_ (Weir and Cockerham 1984) using the hierfstat package (Goudet 2005; Weir and Goudet 2017). Second, we scored whether each study accounted for neutral population structure and/or demography when identifying putatively adaptive SNPs (see Supporting Information File A for more details). Different tools, methods or approaches used to identify putatively adaptive variants vary in how they deal with factors such as demographic history, population structure, non‐independence among populations due to co‐ancestory, spatial autocorrelation, and sharing common gene flow history, all of which can impact false‐positives and negatives depending on the scenario (Excoffier et al. 2009; Lotterhos and Whitlock 2014; Hoban et al. 2016; Ahrens et al. 2018; Booker 2023). Since the methods vary to a large degree, it is challenging to compare the various methodological differences used to identify putatively adaptive SNPs in a fair way. Based on literature, we scored each approach as to whether it accounts for aspects of neutral population structure or demography or high false positive rate as no, yes, and depends (based on whether default settings are used or modifications are made). Then, for each study, we assigned values of 0 where none of the approaches used by the study accounted for these factors, 0.5 if some of the approaches accounted for at least some of these factors and 1 if all the approaches accounted for at least some of these factors. We also included the number of genome‐wide SNPs, the number of putatively adaptive SNPs and the proportion of putatively adaptive SNPs. We expected a part‐whole correlation between the proportion of SNPs that were adaptive and the observed prioritization correlation. Last, we tested the effect of detection method type used to identify putatively adaptive SNPs. We grouped studies that used *F*_*ST*_, with or without other methods, into one category and studies that used other methods, for example, genotype‐environment and/or genotype–phenotype association analyses, into another (i.e., *F*_*ST*_ vs. non‐*F*_*ST*_).

We report Spearman correlation coefficients between the observed correlation and the characteristic of interest for continuous variables (study‐wide *F*_*ST*_, and numbers and proportions of SNPs, all log‐transformed) and one‐way ANOVA for categorical variables (population structure correction and detection method). We report both uncorrected (*p*) and FDR corrected (*p*_*c*_) values given six tests (Table 2). Although we had no a priori predictions, we also tested the impact of these characteristics on the observed minus expected correlations (the predictive power of the putatively adaptive SNPs); we report these latter, with commentary, in the Supporting Information (see Table S1).

All analyses were performed in R version 4.1.1 (R Core Team 2021) unless otherwise stated. We used tidyverse (Wickham et al. 2019), dplyr (Wickham et al. 2022), naniar (Tierney et al. 2021), vcfR (Knaus and Grünwald 2016; Knaus and Grünwald 2017), SNPRelate (Zheng et al. 2012), AssocTests (Wang et al. 2020), adegenet (Jombart 2008; Jombart and Ahmed 2011), spaa (Zhang 2016), ggplot2 (Wickham 2016), cowplot (Wilke 2020), png (Urbanek 2013), vegan (Oksanen et al. 2020), reshape2 (Wickham 2007), dartR (Gruber et al. 2018), stats (R Core Team 2021), palmerpenguins (Horst et al. 2020), rphylopic (Gearty et al. 2023), ggpubr (Kassambara 2020), patchwork (Pedersen 2022), gridExtra (Auguie 2017), and dunn.test (Dinno 2017) packages for data wrangling, visualization, and analysis. We used the Beluga and Cedar Compute Canada clusters (operated by the Digital Research Alliance of Canada) to run all computationally‐intensive analyses.

## 3 Results

We set out to answer the three clear questions posed in the Introduction with implications for the potential use of genetics in population prioritization in conservation and resource management.

Information on the studies we used can be found in Table 1. In total, our final dataset consisted of 34 studies from 35 publications. Our dataset included 2 bird, 8 gymnosperm, 6 angiosperm, 9 fish, 4 mammal, 1 amphibian, and 4 arthropod studies, and the filtered datasets had between 187 and 105,000 genome‐wide SNPs, with between 0.2% to 44% of these being marked as putatively adaptive. Our dataset includes one study that combined population data across two named sister species (Royer et al. 2016), one study that combined data from a three‐species complex (Li et al. 2021), and one study that reported data separately for two named species (Mosca et al. 2016).

**TABLE 1:** Information on studies included in our analyses.

Study number	Study ID	Organism	Number of genome‐wide SNPs	Number of putatively adaptive SNPs	Number of individuals	Number of populations	Method type
1	Ruegg et al. (2018, 2021)	*Empidonax traillii*	105,000	179	144	6	GEA
2	Eimanifar et al. (2018)	*Apis mellifera* L.	2449	140	471	29	*F*_*ST*_
3	White et al. (2013)	*Myodes glareolus*	4340	17	241	14	GEA
4	Schweizer et al. (2016)	*Canis lupus*	13,092	34	106	6	GEA
5	Mosca et al. (2016) *P. mugo*	*Pinus mugo*	615	89	622	20	*F*_*ST*_, GEA
6	McKown et al. (2014), Geraldes et al. (2014)	*Populus trichocarpa*	29,535	410	439	25	GPA
7	Royer et al. (2016)	*Y. brevifolia, Y. jaegeriana*, hybrids	4603	35	197	6	GPA
8	Christmas et al. (2016)	*Dodonaea viscosa*	7255	59	87	17	*F*_*ST*_, GEA
9	Benestan et al. (2016)	*Homarus americanus*	3395	7	421	19	*F*_*ST*_
10	Roffler et al. (2016)	*Ovis dalli dalli*	187	57	472	15	*F*_*ST*_
11	Babin et al. (2017)	*Anguilla rostrata*	12,098	269	710	13	GEA
12	Guo et al. (2016)	*Bufo andrewsi*	15,577	586	264	11	*F*_*ST*_, GEA
13	De Kort et al. (2014)	*Alnus glutinosa*	1714	16	295	23	*F*_*ST*_, GEA
14	Hurel et al. (2021)	*Pinus pinaster Aiton*	6074	96	515	33	GPA
15	Depardieu et al. (2021)	*Picea glauca* [Moench] Voss	6153	359	1473	43	GPA, GEA
16	Chen et al. (2012)	*Picea abies*	375	32	262	18	*F*_*ST*_, GEA
17	Xuereb et al. (2022)	*Oncorhynchus kisutch*	9683	119	836	26	GEA
18	Holliday et al. (2010)	*Picea sitchensis*	437	35	407	13	GPA
19	Flanagan et al. (2021)	*Syngnathus scovelli*	2738	312	235	7	*F*_*ST*_, GEA
20	Bay et al. (2018)	*Setophaga petechia*	104,385	1609	173	19	GEA
21	Chávez‐Galarza et al. (2013)	*Apis mellifera iberiensis*	434	73	668	23	*F*_*ST*_, GEA
22	Keller et al. (2018)	*Populus balsamifera* L.	279	124	925	83	*F*_*ST*_, GEA
23	Funk et al. (2016)	*Urocyon littoralis*	2498	176	103	6	*F*_*ST*_, GEA
24	Mosca et al. (2016) *P.cembra*	*Pinus cembra*	443	85	662	18	*F*_*ST*_, GEA
25	Candy et al. (2015)	*Thaleichthys pacificus*	3725	157	441	11	*F*_*ST*_
26	Dallaire et al. (2021)	*Salvelinus alpinus*	13,596	732	545	23	*F*_*ST*_, GEA
27	Hess et al. (2013)	*Entosphenus tridentatus*	4439	164	513	21	*F*_*ST*_, GPA
28	Milano et al. (2014)	*Merluccius merluccius*	380	30	849	19	*F*_*ST*_, GEA
29	Swaegers et al. (2015)	*Coenagrion scitulum*	3470	566	161	10	*F*_*ST*_, GEA
30	Moore et al. (2014)	*Salmo salar*	3192	374	1079	50	*F*_*ST*_
31	Mahony et al. (2020)	*Pinus contorta*	26,431	460	243	22	GPA
32	He et al. (2016)	*Banksia attenuate*	5701	1049	80	9	*F*_*ST*_
33	Li et al. (2021)	*C.davidi, C. kishinouyei, C. longibarbatus*	5834	258	183	9	*F*_*ST*_
34	Cullingham et al. (2014)	*Pinus banksiana*	361	25	100	4	*F*_*ST*_, GEA

*Note:* *F*_*ST*_ refers to outlier‐type methods, GEA refers to genotype‐environment analyses and GPA refers to genotype–phenotype analyses. Full citations can be found in References list.

**FIGURE 2:**
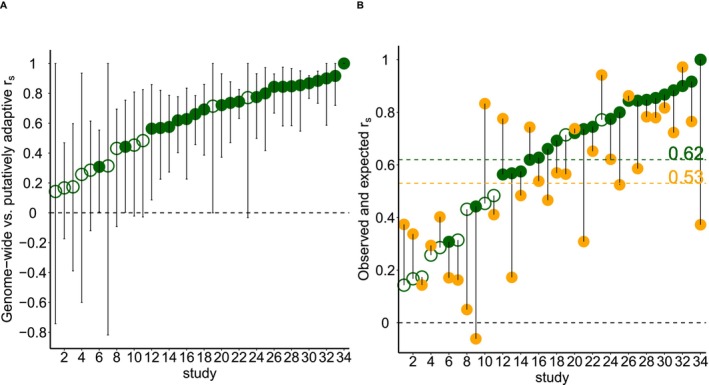
(A) The observed Spearman correlation coefficients (green points) and 90% bootstrapped confidence intervals (black lines) of population distinctiveness scores between genome‐wide and putatively adaptive SNPs (*n* = 34 studies). A more positive value indicates a higher prioritization agreement between the two sets of SNPs. The hollow green points represent cases where 90% bootstrapped confidence intervals of the observed correlations overlap zero. (B) Observed (green, as in (A)) and expected (orange) Spearman correlations of population distinctiveness scores. The expected correlation for each study compares random subsets (1000 bootstrapped replicates) of genome‐wide SNPs with the full genome‐wide set. The vertical black lines for each study represent the predictive power of the putatively adaptive SNPs (see text). The average observed correlation coefficient across all 34 studies (green dashed horizontal line) is higher than the average expected correlation coefficient across all 34 studies (orange dashed horizontal line).

Across all studies, genome‐wide (all) SNPs and putatively adaptive SNPs tended to provide similar population rankings. We found positive correlations between the prioritization rankings based on both sets of SNPs for all studies and for 23/34 of these the 90% confidence intervals did not include 0 (Figure 2A). The observed Spearman correlation coefficients varied from +0.14 to +1.00 with a mean *r*_*s*_ of 0.62 across studies. When we subset the dataset to only include studies with the largest datasets (> 1000 SNPs), this relationship remained consistent: Spearman correlation coefficients varied from +0.14 to +0.92 with a mean *r*_*s*_ of 0.59; see Figure S7.

Putatively adaptive SNPs are a subset of genome‐wide SNPs, leading to a part‐whole correlation. Given this, we compared the observed correlation with the ‘expected correlation’ using random subsets of SNPs, that is, samples with the same expected part‐whole correlation structure. A weaker‐than‐expected correlation would support the contention that putatively adaptive SNPs show different patterns than the entire genome. Unexpectedly, we found that putatively adaptive subsets of SNPs show a stronger‐than‐expected correlation in two thirds of all cases (Figure 2B). A two‐tailed paired *t*‐test confirmed this difference (*t*(33) = 2.45, *p* = 0.02) with a mean difference of 0.09 (95% CI: 0.02 to 0.17). This difference remained after removing three outlier studies (that fell above the Q3 + 1.5xIQR or below Q1–1.5xIQR) using the ‘identify_outliers’ function from the rstatix package (Kassambara 2023): *t*(30) = 2.48, *p* = 0.02 with a mean difference of 0.08.

We note that methods used to identify adaptive SNPs are more likely to identify SNPs at intermediate allele frequencies (Lasky et al. 2022), so putatively adaptive SNPs may be at higher average frequency compared to non‐adaptive SNPs overall. This might increase their ability to reflect species‐wide patterns. Indeed, putatively adaptive SNPs were often found at higher average frequencies (Figure S5; mean difference between median allele frequency of adaptive and median allele frequency of size‐matched random sets of genome‐wide SNPs = 0.07, 95% CI: 0.04 to Inf, one‐tailed *t*(33) = 4.42, *p* < 0.001), though the degree to which they deviate from overall SNP frequency does not predict their ability to track genome‐wide variation at all (Figure S6, Spearman correlation (*r*_*s*_): −0.12, *p* = 0.50).

By using the data reported by the original studies and the SNPs they identified as adaptive, we tested whether six different characteristics of the datasets we tested were related to agreement in the priority rankings (Table 2). As expected, the proportion of all SNPs that were putatively adaptive predicted the prioritization agreement (Table 2): the larger this fraction the more the genome‐wide SNP rankings will reflect the data in the putatively adaptive category. None of the other characteristics independently predicted prioritization agreement (see also Supporting Information). When we subset the dataset to only include studies with the largest datasets (> 1000 SNPs), the log of number of putatively adaptive SNPs also emerged as significant; after further regression analysis, we found that the proportion of putatively adaptive SNPs drives this correlation (see Supporting Information).

**TABLE 2:** Impact of six characteristics on the prioritization agreement. *r*_*s*_ is Spearman rank correlation coefficient, *p* is the uncorrected *p*‐value and *p*_*c*_ is the FDR‐corrected *p*‐value.

Study‐based characteristics	Prioritization agreement
Log (study‐wide *F*_*ST*_)	*r*_*s*_ = −0.01; *p* = 0.94; *p*_*c*_ = 0.94
Account for neutral population structure or demography	*p* = 0.56; *p*_*c*_ = 0.67
Log (number of genome‐wide SNPs)	*r*_*s*_ = −0.20; *p* = 0.26; *p*_*c*_ = 0.39
Log (number of putatively adaptive SNPs)	*r*_*s*_ = 0.32; *p* = 0.06; *p*_*c*_ = 0.13
Log (proportion of putatively adaptive SNPs)	*r*_*s*_ = 0.50; *p* = 0.003; *p*_*c*_ = 0.02
Method type (*F*_*ST*_ vs. non *F*_*ST*_)	*p* = 0.02; *p*_*c*_ = 0.05

## 4 Discussion

Overall, we found clear evidence that putatively adaptive and genome‐wide genetic distinctiveness provide similar patterns. The methods and datatypes used in previous studies have varied, but similar findings have been reported for sage grouse (Oh et al. 2019), Atlantic salmon (Moore et al. 2014), coho salmon (Xuereb et al. 2022), Eastern Massasauga rattlesnake (Mathur et al. 2023), maize (Li et al. 2025), and several species of hermaphroditic plants (Bataillon et al. 1996).

Our findings are relevant to genetic resource and conservation management for both plants and animals. For example, the debate in the forestry research community on the need to identify adaptive genetic variation remains unresolved (Lind et al. 2018; Fady et al. 2020). Current methods to identify putatively adaptive alleles, including genome‐wide association studies (GWAS), seem to have limited power to predict phenotypic variation (Li et al. 2025; Slavov et al. 2025). Management decisions require careful consideration of specific risks and benefits on a case‐by‐case basis, and our results support a focus on genome‐wide genetic variation in many applied contexts (see also Lind and Lotterhos 2024).

The debate on the use of putatively adaptive genomic variation in conservation prioritization is decades old. Given the current limitations and challenges in identifying and using adaptive variation for conservation, genome‐wide variation, which is much easier to measure, has been considered a useful surrogate for adaptive variation (see DeWoody et al. 2021; García‐Dorado and Caballero 2021; Kardos et al. 2021). That said, both theoretical and empirical evidence for congruence remains mixed (for congruence, see, e.g., Merilä and Crnokrak 2001, Leinonen et al. 2008, Moore et al. 2014, Oh et al. 2019, Xuereb et al. 2022, Mathur et al. 2023, Li et al. 2025; for noncongruence, see Reed and Frankham 2001, Reed and Frankham 2003, Mittell et al. 2015, Pecoraro et al. 2018, Sandoval‐Castillo et al. 2018, Teixeira and Huber 2021, Xuereb et al. 2021). Across our 34 studies, we found that genome‐wide and putatively adaptive subsets of genome‐wide genetic distinctiveness generally agree on conservation prioritization, with the strength of agreement mainly driven by the proportion of putatively adaptive SNPs.

To the extent that adaptation is polygenic (Rockman 2012; Bertin et al. 2020), this part‐whole correlation supports the use of genome‐wide genetic variation for prioritization when using genetics for conservation. As others have, we emphasize that more work is needed to catalog the extent to which adaptation is polygenic vs. when it is governed by few genes of large effect (Bomblies and Peichel 2022).

An alternative explanation for our general finding is that outlier scans may not illuminate the genetic basis of adaptation, and putatively adaptive genes are not indeed adaptive (i.e., are false‐positives). This would also explain why our measure of population structure (*F*_*ST*_) does not predict congruence (others are outlined in the Supporting Information).

At face value, the congruence we report is consistent with adaptation being highly polygenic, such that our genome‐wide variation includes many SNPs associated with adaptive alleles of small effect. Genome‐wide and adaptive loci may also be subjected to similar evolutionary processes leading to similar patterns (Moore et al. 2014; Pearse 2016; Kardos et al. 2021). In particular, divergent selection regimes causing lower gene flow may lead to neutral genomic divergence as a by‐product due to linkage among neutral and non‐neutral loci (Merilä and Crnokrak 2001; Leinonen et al. 2008; Nosil et al. 2008; Nosil et al. 2009). To the extent that polygenic adaptation is common (Rockman 2012; Harrisson et al. 2014; Sella and Barton 2019; Booker et al. 2020; Capblancq et al. 2020; Fernandez‐Fournier et al. 2021; Hayward and Sella 2022; Kowalczyk et al. 2022), all this would increase the observed correlation and support the use of genome‐wide variation for conservation prioritization.

### 4.1 Limitations

There is likely a lot of noise in our dataset: there are limitations with current outlier differentiation and association analyses used to detect putatively adaptive loci, limitations that likely bias studies towards identifying large effect loci (Harrisson et al. 2014; Hoban et al. 2016). There are also many confounding factors including population structure, demography, high gene flow, covariance among alleles due to coancestry among populations, the possibility that the neutral isolation by distance gradient aligns with selection gradients, correlations among climatic and geographic distances with shared population history, incorrect demographic models, and selection effects. Importantly, these confounding factors can lead to both false‐negative and false‐positive inference of adaptive loci (Aitken and Whitlock 2013; Sork et al. 2013; Lotterhos and Whitlock 2014; Ahrens et al. 2018; Forest et al. 2018; Dallaire et al. 2021).

Because some of our studies are from more than a decade ago, when SNP densities were lower, we reran the analyses with only the studies with more than 1000 SNPs. Our main findings were consistent.

### 4.2 Future Directions

Alongside threat status, risk assessment and costs, the prioritization of conservation activity based on genetic patterns is likely to grow in importance (Fernandez‐Fournier et al. 2021). Improvements to the genetic metrics used to prioritize populations in this study will be welcome. In particular, we may need to account for both within‐and among‐population genetic variation simultaneously, particularly as it has been shown that more distinct populations may harbour lower intra‐population variation (Coleman et al. 2013; Weeks et al. 2016). Many studies (Hard 1995; Petit et al. 1998; Pannell and Charlesworth 2000; Caballero and Toro 2002; Coart et al. 2005; Toro and Caballero 2005; Pérez‐Figueroa et al. 2009; Taylor et al. 2011; Coleman et al. 2013; Li et al. 2018; Hoban et al. 2020; Laikre et al. 2020) have advocated, developed or implemented methods to harmonize both within‐and among‐population genetic distinctiveness: one simple approach that draws on the Shapley value and the framework presented by Abhari and colleagues (Abhari et al. 2024) focuses on a population's expected contribution to total pooled heterozygosity, introduced in Chhina et al. (2024).

It is very challenging to measure the extent to which population distinctiveness is driven by local adaptation versus genetic drift (Fernandez‐Fournier et al. 2021), with serious conservation management implications (Ottewell et al. 2016; Liddell et al. 2021). Because of this, it is important for managers to consider other measures when assessing the conservation value of any particular population (Capmourteres and Anand 2016). That said, and most generally, if distinct populations exhibit multiple signatures of local adaptation and are not at risk of imminent extinction, then managing them separately might be suitable (Fraser and Bernatchez 2001; Aitken and Whitlock 2013), e.g., as advocated for the Halfmoon Hairstreak butterfly (COSEWIC 2022; MacDonald et al. 2025). However, if populations are small and declining where drift may be a strong force, managing the populations separately is likely to be detrimental overall (Höglund 2009; Assis et al. 2013; McMahon et al. 2014; Volkmann et al. 2014; Weeks et al. 2016). In these cases, promoting gene flow (alongside increasing population sizes) might be beneficial (Aitken and Whitlock 2013; Weeks et al. 2016; Ralls et al. 2018), for e.g., as advocated for the Yellow‐tufted honeyeater ( *Lichenostomus melanops cassidix* ) (Harrisson et al. 2016; Pavlova et al. 2023). Chhina et al. (2024) offer further examples of each situation.

We propose that the community standardize methodologies for collecting and reporting SNP data, and for identifying putatively adaptive SNPs. This might include agreement on SNP naming, missing rate cut‐offs, significance thresholds and the consideration of neutral population structure, as well as standardized data formats (e.g., VCF), and access to geolocation data. Increased overall sample sizes to allow for biological characteristics to be considered would also be beneficial. Some of the challenges in reproducibility, interpretation, validation (Forester et al. 2022), and fair comparison within and across studies can be solved via standardization of study properties.

Earlier work in conservation genomics highlighted the limitations of low SNP density for detecting adaptive variation, as reduced marker coverage can limit statistical power and the ability to capture loci linked to selection (e.g., Morin et al. 2009; Smouse 2010; Landguth et al. 2012). However, many studies included in this project utilised powerful, efficient, and cost‐effective RADseq and genotyping‐by‐sequencing methods, which are widely applied, easy to navigate by investigators (Parchman et al. 2018; de Ronne et al. 2023), and these have substantially increased the number of loci available. Future studies could further improve our ability to detect adaptive variation by harnessing whole genome sequencing, enabling more complete discovery across the full genome and higher analytical power (Theissinger et al. 2023). Whether such approaches can be applied broadly to the many taxa of conservation concern is a question of resources.

Structural variants (SVs), such as inversions, copy‐number variants, and translocations, can capture large‐effect genomic variation that is missed by SNP‐based approaches. Incorporating SV data can therefore improve the delineation of conservation units by revealing additional adaptive differentiation and genomic regions under selection. This may be particularly useful in taxa with complex genomic architecture and strong local adaptation. However, SV genotyping and analytical methods are still an emerging area of research in population genetics (Wold et al. 2023; Stuart et al. 2026), and so uptake by conservation practitioners (at least in the short‐term) is likely to be limited due to capacity and budgetary constraints. In addition, there are detection, profiling, genotyping, and analytical biases that need to be overcome before SV data can contribute to conservation geneticists at scale (Stuart et al. 2026).

## 5 Conclusion

Based on the overall agreement across the 34 studies considered here, practitioners should consider using genome‐wide genetic variation when using genetics for prioritizing populations. We note that the biology, the data, and the methods for identifying putatively adaptive genetic variation varied greatly across studies. While this might seem a strength, it does limit fair comparisons among studies. Methods to identify true adaptive variants are still improving, but it seems reasonable to assume the datasets we considered here include many putatively adaptive variants that are not truly adaptive, while many others were missed. Given all the caveats associated with identifying adaptive loci and the fact that current approaches identify loci that are only currently adaptive (Allendorf et al. 2010; Shafer et al. 2015; Bertin et al. 2020; García‐Dorado and Caballero 2021) in the context of rapidly changing ecologies where selection coefficients can change rapidly, we believe it may be prudent to consider as much genome‐wide variation as possible when doing conservation genetics. Identifying genetically distinctive populations can contribute to this endeavour.

## Author Contributions

A.K.C. and A.M. co‐wrote the first draft of the manuscript. A.K.C. collated the datasets. A.K.C., P.F.‐F., J.M.M.L., A.M., and T.R.B. analyzed the data. All authors revised the manuscript.

## Funding

Funding for this work was provided by NSERC (RGPIN‐2019‐04950, RGPIN‐2025‐04375 and RGPIN‐2024‐04434) Discovery Grants awarded to A.M. and T.R.B. A.K.C. was supported by graduate scholarships from NSERC, the BC government, and Simon Fraser University. P.F.F. acknowledges graduate funding from NSERC and Simon Fraser University, and J.M.M.L. acknowledges the Liber Ero Fellowship Program.

## Conflicts of Interest

The authors declare no conflicts of interest.

## Supporting information


**Figure S1:** Proportion of putatively adaptive SNPs positively impacts the prioritization agreement (genome‐wide vs. putatively adaptive *r*_*s*_) as expected by the part‐whole regression. Each dark green point represents a study (*n* = 34). The Spearman correlation (*r*_*s*_) between the prioritization agreement and log(proportion of putatively adaptive SNPs) is 0.50 (uncorrected *p* = 0.003, FDR corrected *p*_*c*_ = 0.02).
**Figure S2:** Method‐type used to identify putatively adaptive SNPs seems to impact the prioritization agreement. One‐way ANOVA uncorrected *p* = 0.02, FDR corrected *p*_*c*_ = 0.05 (*n* = 34).
**Figure S3:** Increasing study‐wide *F*_*ST*_ seems to decrease the predictive power of putatively adaptive SNPs relative to random expectation. Each green point represents a study (*n* = 34). The Spearman correlation (*r*_*s*_) is −0.46 with uncorrected *p* = 0.007; FDR corrected *p*_*c*_ = 0.04.
**Figure S4:** Increasing number of putatively adaptive SNPs seem to decrease the predictive power of putatively adaptive SNPs relative to random expectation. Each green point represents a study (*n* = 34). The spearman rho correlation (*r*_*s*_) is −0.37, uncorrected *p* = 0.03, FDR corrected *p*_*c*_ = 0.09.
**Figure S5:** Median minor allele frequencies of putatively random (orange) and adaptive (green) SNPs for each study (*n* = 34). The black line indicates the difference between median allele frequencies of putatively adaptive and size‐matched random (initial seed = 12) subsets of genome‐wide SNPs. Asterisks for 19 out of 34 studies represent statistical significant differences (alpha = 0.05, uncorrected) between median minor allele frequencies of putatively adaptive and random subsets of genome‐wide SNPs based on a Mann–Whitney *U* test.
**Figure S6:** Scatterplot showing the predictive power of putatively adaptive SNPs (i.e., observed minus expected *r*_*s*_) on y‐axis and median adaptive minus random allele frequencies for each study (*n* = 34) on x‐axis. There is no significant correlation between the two (Spearman rho (*r*_*s*_) = −0.12 with *p* = 0.50).
**Figure S7:** (A) The observed Spearman correlation coefficients (green points) and 90% bootstrapped confidence intervals (black lines) of population distinctiveness scores between genome‐wide and putatively adaptive SNPs for a subset of studies with > 1000 SNPs (*n* = 25 studies). A more positive value indicates a higher prioritization agreement between the two sets of SNPs. The hollow green points represent cases where 90% bootstrapped confidence intervals of the observed correlations overlap zero. (B) Observed (green, as in (A)) and expected (orange) Spearman correlations of population distinctiveness scores. The expected correlation for each study compares random subsets (1000 bootstrapped replicates) of genome‐wide SNPs with the full genome‐wide set. The vertical black lines for each study represent the predictive power of the putatively adaptive SNPs (see text).
**Figure S8:** No relationship between number of individuals per population in our final dataset and the correlation between genome‐wide and putatively adaptive SNPs. Note that all populations with fewer than 4 individuals were not included in our final dataset in any analysis.
**Table S1:** Impact of six characteristics on the prioritization agreement and the predictive power of putatively adaptive SNPs. Asterisk (*) indicates a *p* < 0.05 (uncorrected for FDR).
**Table S2:** The table provides the correlation among all six characteristics. Some study‐based characteristics co‐vary and show high correlation such as the proportion of putatively adaptive SNPs, method‐type, and number of genome‐wide SNPs.
**Table S3:** Impact of six characteristics on the prioritization agreement and the predictive power of putatively adaptive SNPs using only the 25 studies with more than 1000 SNPs. *r*_*s*_ is the Spearman correlation, *p* is uncorrected *p*‐value, *p*_*c*_ is FDR‐corrected *p* value.

**Data S1:** mec70483‐sup‐0002‐Supinfo1.zip.

## Data Availability

All data and analysis code can be found on Dryad: https://doi.org/10.5061/dryad.nvx0k6f1j. Additional files accompanying this article include: File A includes information on whether the type of analysis used to detect putatively adaptive SNPs accounted for neutral population structure or impact of population demography or contributed to reduction in high false‐positives. File A. Details_on_accounting_for_neutral_population_structure_or_false_positives_file.csv File B contains information on 26 variables for 34 studies and is used as the input file for Files C and D. File B. Details_of_34_studies.csv. File C contains the code for all figures and tables as well as meta statistical analysis performed on the data using 34 studies. File C.R_script_for_tables_figures_and_statistical_analysis.R. File D contains the code for the re‐analysis using only the subset of the dataset (25 studies). File D. R script for reanalysis using subset of the dataset R.
