# Supplementary material for: Putatively Adaptive and Genome‐Wide Genetic Variation Often Rank Populations as Similarly Distinctive
Source: Mol Ecol. 2026 Aug 4;35(15):e70483. doi: 10.1111/mec.70483 (PMC13435827; doi:10.1111/mec.70483)
Supplement: Supplementary file 1 [file MEC-35-e70483-s001.pdf]

Putatively adaptive and genome-wide genetic variation often rank populations as similarly distinctive

Avneet K. Chhina[1,*], Philippe Fernandez-Fournier[1], Jayme M.M. Lewthwaite[2,3], Tom R. Booker[4], and Arne Mooers[1,*]

[1]Department of Biological Sciences, Simon Fraser University, BC, Canada

[2]Department of Biology, Carleton University, Ottawa, ON, Canada

[3]Department of Entomology, Cornell University, Ithaca, NY, USA

[4]Department of Forest and Conservation Sciences, University of British Columbia, BC, Canada

[*]Corresponding authors:Avneet K. Chhina (chhina.k.avneet@gmail.com) and Arne Mooers (amooers@sfu.ca)

1 Supplementary material

1.1 Study-based characteristics

1. Study-wide F_{ST}. If F_{ST} is affected by local adaptation, then one would expect adaptive allele variation among populations to mirror genome-wide patterns dictated by concomitant low gene flow. However, F_{ST} may not be mediated by local adaptation, such that we would see no effect of F_{ST}. Alternatively or in addition, our putatively adaptive SNPs may include many false positives,

also weakening the effect of F_{ST} on the correlation. False positives would inflate prioritization agreement but if there is high differentiation among populations it may be easier to detect the genes involved in local adaptation (Savolainen et al. 2007; Booker 2023) leading to fewer of those false positives. Additional analysis (for interest purposes) showed no relationship (r_s = 0.02; p = 0.91) between number of individuals sequenced per population in our final dataset and the correlation between genome-wide and putatively adaptive SNPs, as shown in the Figure S8. Note that all populations with fewer than 4 individuals were not included in our final dataset in any analysis.

2. Whether the study corrected for neutral population structure. Non-independence among populations due to coancestry (reflecting shared population history) (Bonhomme et al. 2010), spatial autocorrelation (ex. isolation-by-distance, environment or more), shared common gene flow history or demographic history (see Lotterhos and Whitlock 2014; Hoban et al. 2016; Booker 2023) could all impact the correlation between genome-wide loci and loci under selection. Importantly, these patterns can inflate false-negatives as well as false-positive identification of adaptive SNPs, depending on the scenario (Günther and Coop 2013; Lotterhos and Whitlock 2014; Ahrens et al. 2018). Some types of analysis estimate and correct for evolutionary non-independence among samples during the process of identification of adaptive SNPs whereas others do not (Beaumont and Nichols 1996; Excoffier et al. 2009; Cullingham et al. 2014; Lotterhos and Whitlock 2014; see File A for more details).

3-5. Number of genome-wide SNPs, number of putatively adaptive SNPs, proportion of putatively adaptive SNPs. These quantities reflect aspects of sampling strategies that might also affect the power to differentiate adaptive from non-adaptive loci (Ahrens et al. 2018). If the dataset size or the number of putatively adaptive SNPs impact the correlation, this would suggest that caution should be applied with the interpretation of results (Ahrens et al. 2018). We note that we chose not to analyze the correlation between putatively adaptive SNPs and putatively non-adaptive SNPs because this latter fraction would not

be part of the conservation genomics datastream we are interested in; if putatively adaptive SNPs have been identified for conservation, the investment has been made.

6. The detection method type (F_{ST} vs. non-F_{ST}) used to identify putatively adaptive SNPs. We suspected that F_{ST} methods might produce more false positive adaptive SNPs (Sork et al. 2013) than other methods, and that this would inflate prioritization agreement.

We expected a negative correlation between study-wide F_{ST} and prioritization agreement, but found none (Table 2). Using one-way ANOVA, we also found that accounting for neutral population structure did not affect the prioritization agreement in the studies analyzed. We found that neither the number of genome-wide SNPs nor the number of putatively adaptive SNPs impacted prioritization agreement (Table 2). In contrast, and as expected due to the part-whole correlation, the proportion of putatively adaptive SNPs was positively associated with agreement (Spearman correlation (r_s) = 0.50, p = 0.003, p_c = 0.02) (Figure S1). The method-type (F_{ST} vs. non F_{ST}) used to identify putatively adaptive SNPs also impacted the prioritization agreement (p = 0.02, p_c = 0.05), with F_{ST} method showing higher correlation between genome-wide and putatively adaptive SNPs than non-F_{ST} methods in the studies analyzed (Figure S2). Because the proportion of putatively adaptive SNPs differed significantly between F_{ST} vs. non-F_{ST} methods (one-way ANOVA p = 0.0005), we performed a secondary analysis, a simple beta regression (using the `betareg` R-package (Cribari-Neto and Zeileis 2010)) to contrast these two predictors. We found that the log of proportion of putatively adaptive SNPs (partial coefficient = 0.31, p = 0.04, n=34) is the stronger predictor compared to method type (partial coefficient = -0.57, p = 0.20). This holds after removing one outlier study (Cullingham et al. 2014) (proportion of putatively adaptive SNPs: coefficient = 0.33, p = 0.009, n=33; method type: coefficient = -0.21, p = 0.53).

We note that some of the six study-based characteristics considered here themselves co-vary (see Table S2). The proportion of putatively adaptive SNPs

decreased with the total number of genome-wide SNPs (Spearman correlation (r_s) = -0.66, p = 3.13e-05) (pattern consistent with Ahrens et al. 2018); the reasons for this remain unknown. In our sample, the proportion of putatively adaptive SNPs seem to be impacted by the method-type used, being higher for F_{ST} compared to non-F_{ST} methods ($F_{1,32}$ = 15.21, p < 0.001). This result is hard to interpret since methods were not applied in a similar way across studies.

As stated in the main text, we had no a priori predictions for the effect of study-based characteristics on the predictive power of putatively adaptive SNPs. Study-wide F_{ST} was negatively correlated with this measure (Spearman correlation (r_s) = -0.46, p = 0.007, p_c = 0.04): as study-wide F_{ST} increased, the correlation between genome-wide and putatively adaptive SNPs decreased relative to random expectation (Figure S3). The number of putatively adaptive SNPs was also negatively correlated with predictive power of putatively adaptive SNPs (Spearman correlation (r_s) = -0.37, p = 0.03, p_c = 0.09): with higher numbers of putatively adaptive SNPs behave like random SNPs do (Figure S4). Using an ANOVA, we found that both the log(number of putatively adaptive SNPs) (estimate = -0.06, p = 0.009) and log(study-wide F_{ST}) (estimate = -0.05, p = 0.03) affect the predictive power of putatively adaptive SNPs (however, see Section 1.2). None of the other study-based characteristics were associated with predictive power.

Table S1: Impact of six characteristics on the prioritization agreement and the predictive power of putatively adaptive SNPs. Asterisk (*) indicates a p $<$ 0.05 (uncorrected for FDR)

1

Study-based characteristics	Prioritization agreement	Predictive power of putatively adaptive SNPs
log(study-wide F_{ST})	r_s = -0.01, p = 0.94	r_s = -0.46, p = 0.007*
Account for neutral population structure or demography	p = 0.56	p = 0.45
log(number of genome-wide SNPs)	r_s = -0.20, p = 0.26	r_s = -0.17, p = 0.34
log(number of putatively adaptive SNPs)	r_s = 0.32, p = 0.06	r_s = -0.37, p = 0.03*
log(proportion of putatively adaptive SNPs)	r_s = 0.50, p = 0.003*	r_s = -0.17, p = 0.35
Method Type (F_{ST} vs. non-F_{ST})	p = 0.02*	p = 0.38

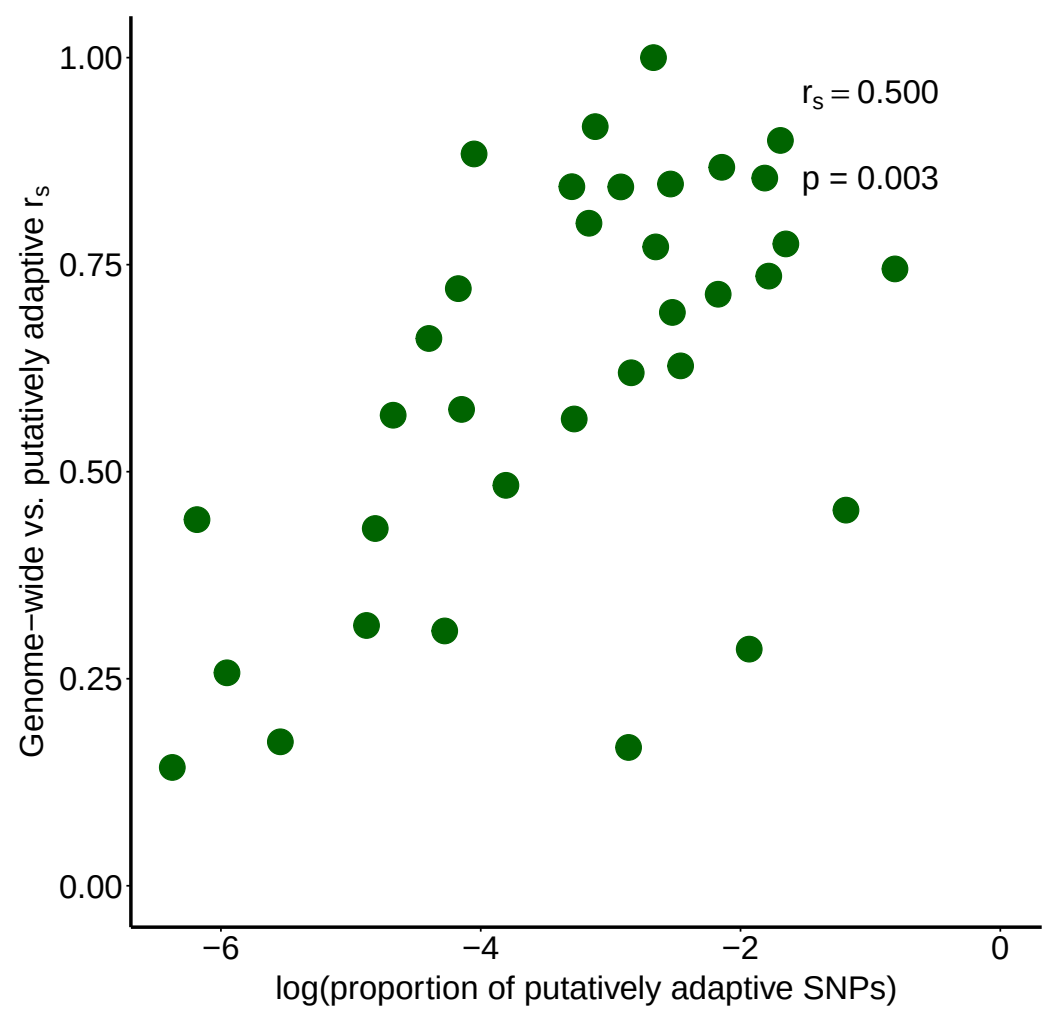

Figure S1: Proportion of putatively adaptive SNPs positively impacts the prioritization agreement (genome-wide vs. putatively adaptive r_s) as expected by the part-whole regression. Each dark green point represents a study (n = 34). The Spearman correlation (r_s) between the prioritization agreement and log(proportion of putatively adaptive SNPs) is 0.50 (uncorrected p = 0.003, FDR corrected p_c = 0.02).

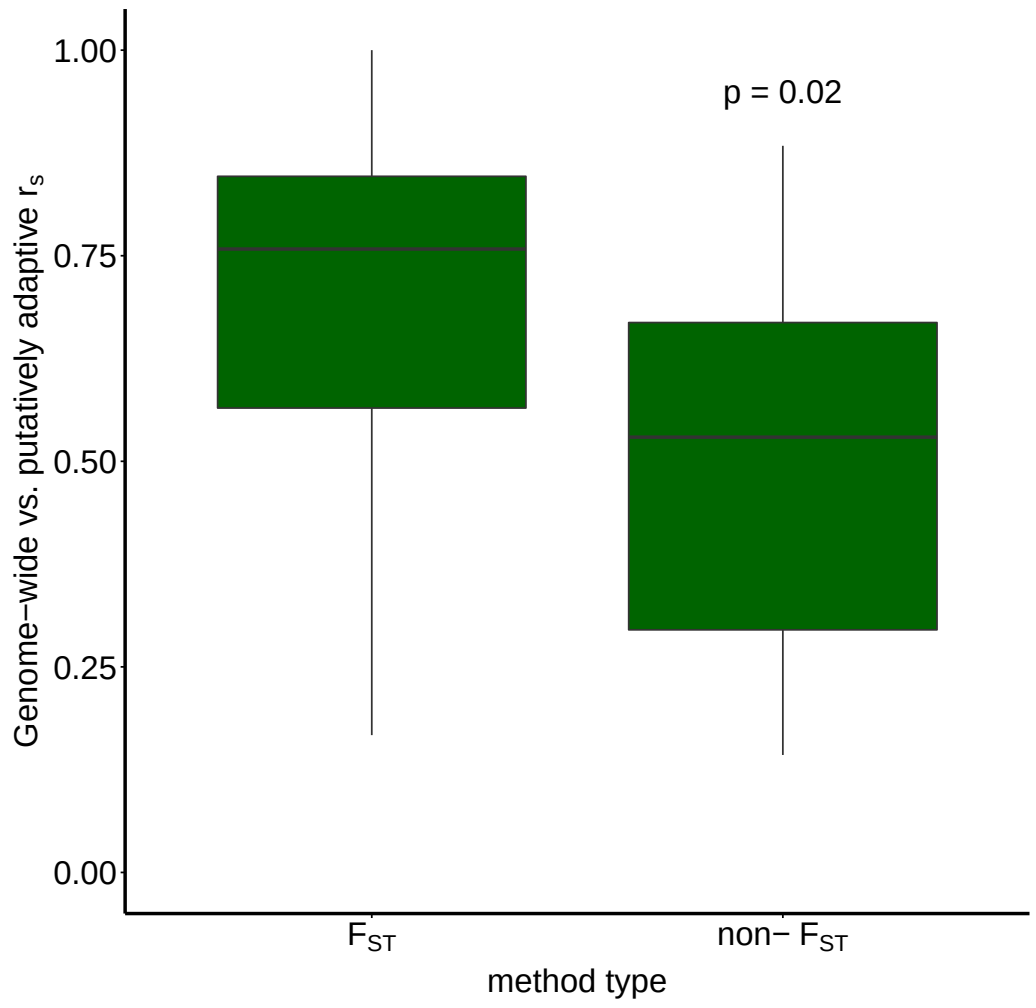

Figure S2: Method-type used to identify putatively adaptive SNPs seems to impact the prioritization agreement. One-way ANOVA uncorrected p = 0.02, FDR corrected $p_c = 0.05$ (n = 34).

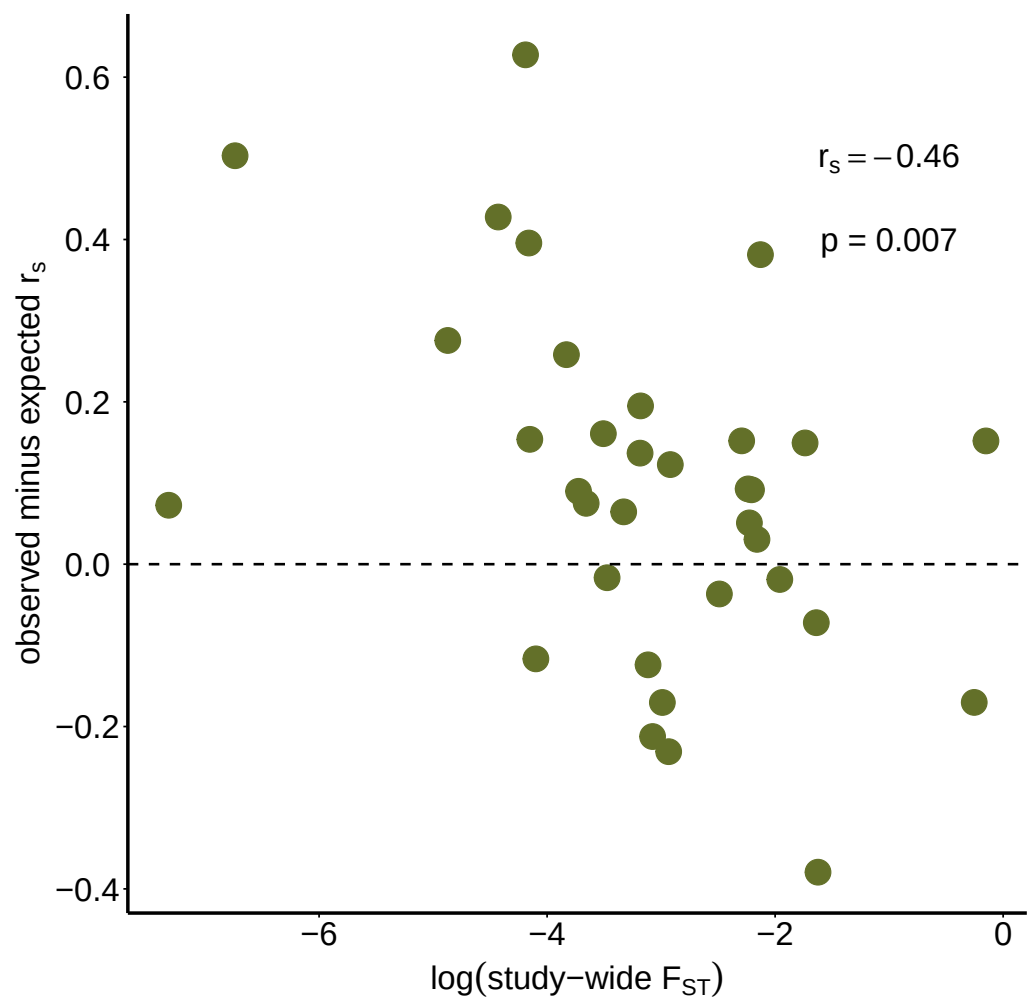

Figure S3: Increasing study-wide F_{ST} seems to decrease the predictive power of putatively adaptive SNPs relative to random expectation. Each green point represents a study (n = 34). The Spearman correlation (r_s) is -0.46 with uncorrected p = 0.007; FDR corrected $p_c = 0.04$.

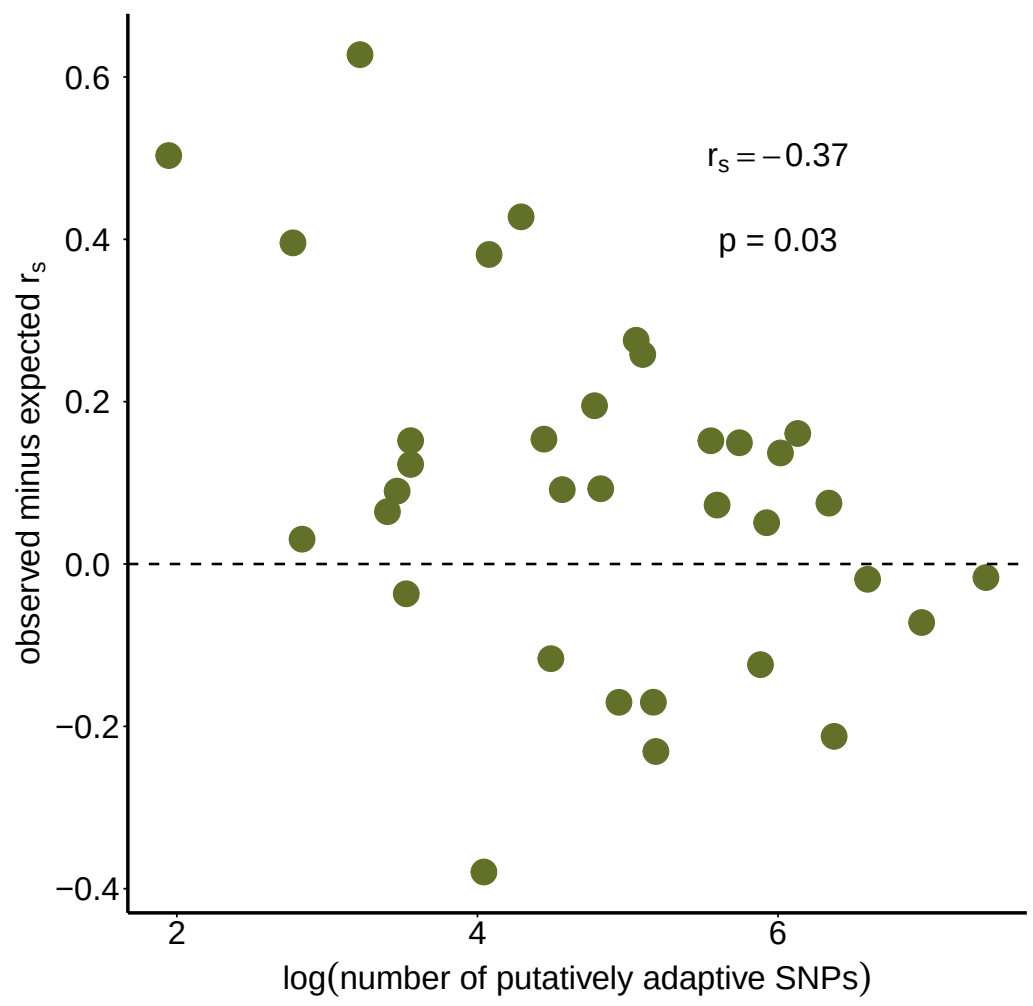

Figure S4: Increasing number of putatively adaptive SNPs seem to decrease the predictive power of putatively adaptive SNPs relative to random expectation. Each green point represents a study (n = 34). The spearman rho correlation (r_s) is -0.37, uncorrected p = 0.03, FDR corrected $p_c = 0.09$.

Table S2: The table provides the correlation among all six characteristics. Some study-based characteristics co-vary and show high correlation such as the proportion of putatively adaptive SNPs, method-type, and number of genome-wide SNPs.

	log(number of putatively adaptive SNPs)	log(proportion of putatively adaptive SNPs)	log(genome-wide SNPs)	log(study-wide F_{ST})	Account for neutral population structure or demography	Method Type (F_{ST} vs. non-F_{ST})
log(number of putatively adaptive SNPs)	1	0.29	0.54	0.24	0.24	0.06
log(proportion of putatively adaptive SNPs)	0.29	1	-0.65	0.18	-0.29	-0.57
log(number of genome-wide SNPs)	0.54	-0.65	1	0.03	0.45	0.55
log(study-wide F_{ST})	0.24	0.18	0.03	1	0.009	-0.06
Account for neutral population structure or demography	0.24	-0.29	0.45	0.009	1	0.55
Method Type (F_{ST} vs. Non-F_{ST})	0.06	-0.57	0.55	-0.06	0.55	1

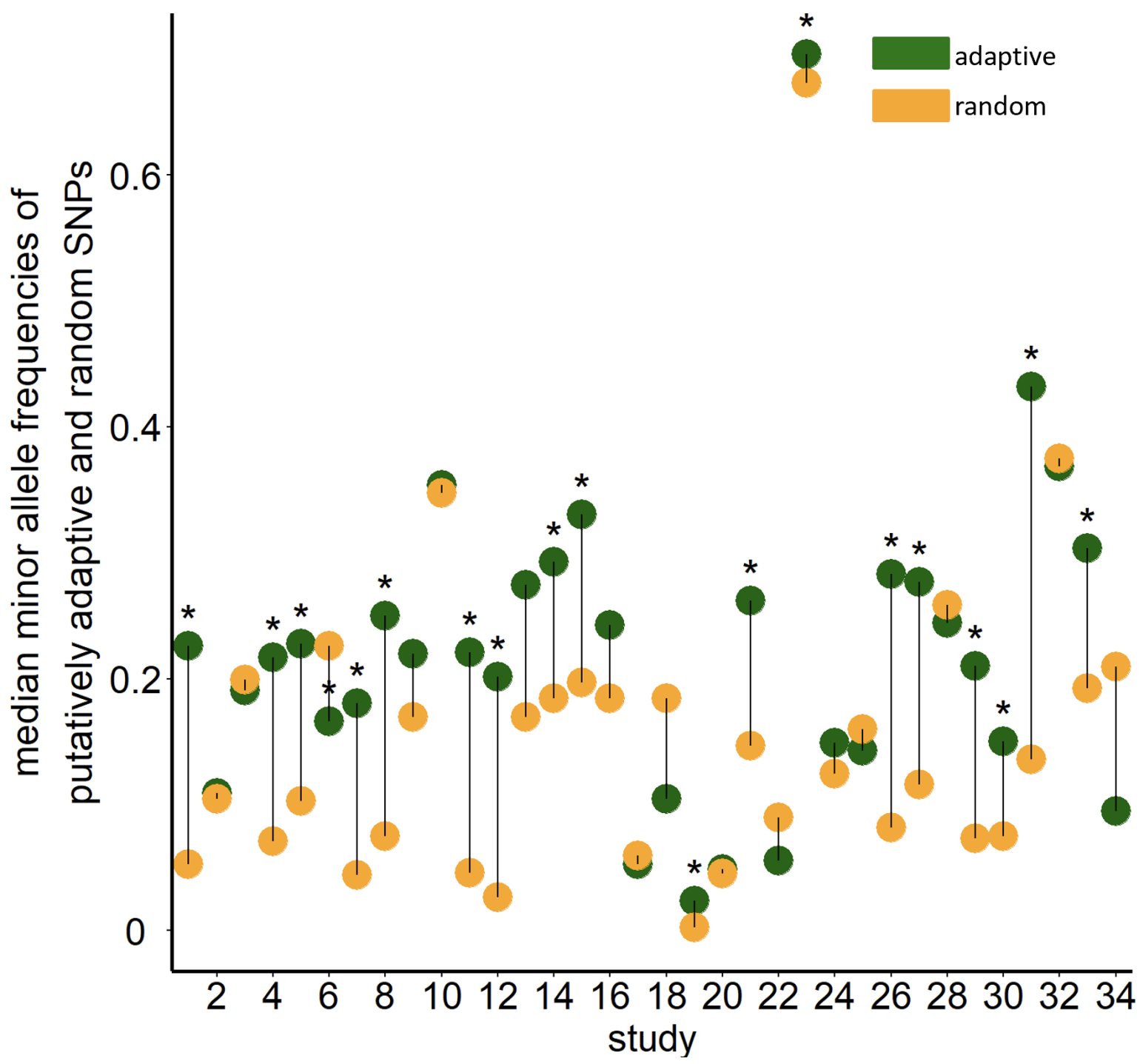

Figure S5: Median minor allele frequencies of putatively random (orange) and adaptive (green) SNPs for each study (n = 34). The black line indicates the difference between median allele frequencies of putatively adaptive and size-matched random (initial seed = 12) subsets of genome-wide SNPs. Asterisks for 19 out of 34 studies represent statistical significant differences (alpha=0.05, uncorrected) between median minor allele frequencies of putatively adaptive and random subsets of genome-wide SNPs based on a Mann-Whitney U test.

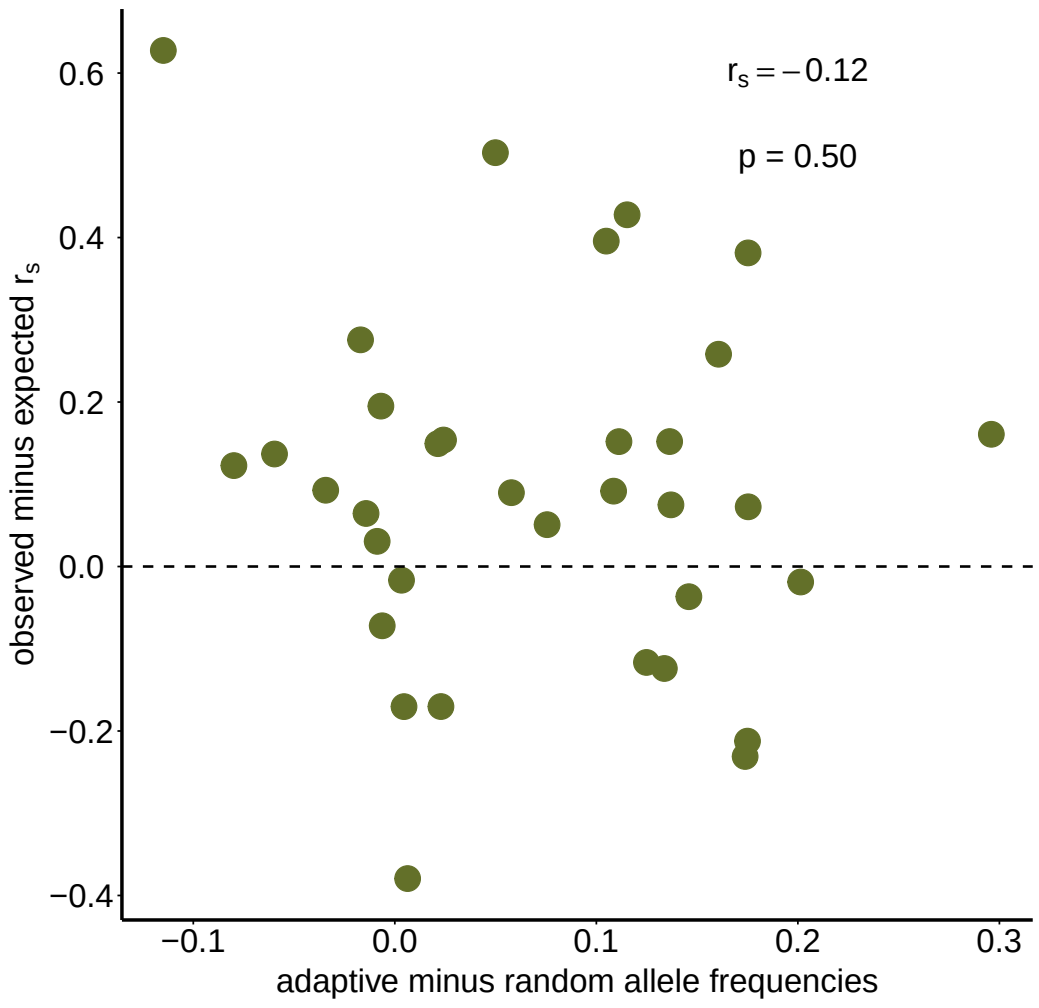

Figure S6: Scatterplot showing the predictive power of putatively adaptive SNPs (i.e., observed minus expected r_s) on y-axis and median adaptive minus random allele frequencies for each study (n= 34) on x-axis. There is no significant correlation between the two (Spearman rho (r_s) = -0.12 with p = 0.50).

1.2 Repeated main analysis with only the 25 studies with more than 1000 genome-wide SNPs

Analyses on small datasets may have high sampling error. We therefore repeated the main analysis using only the 25 studies that contained more than 1000 SNPs. The observed Spearman correlation coefficients varied from +0.14 to +0.92 with a mean r_s of 0.59 across studies; for 16 of the 25 studies, the 90% confidence intervals did not include 0 (Figure S7).

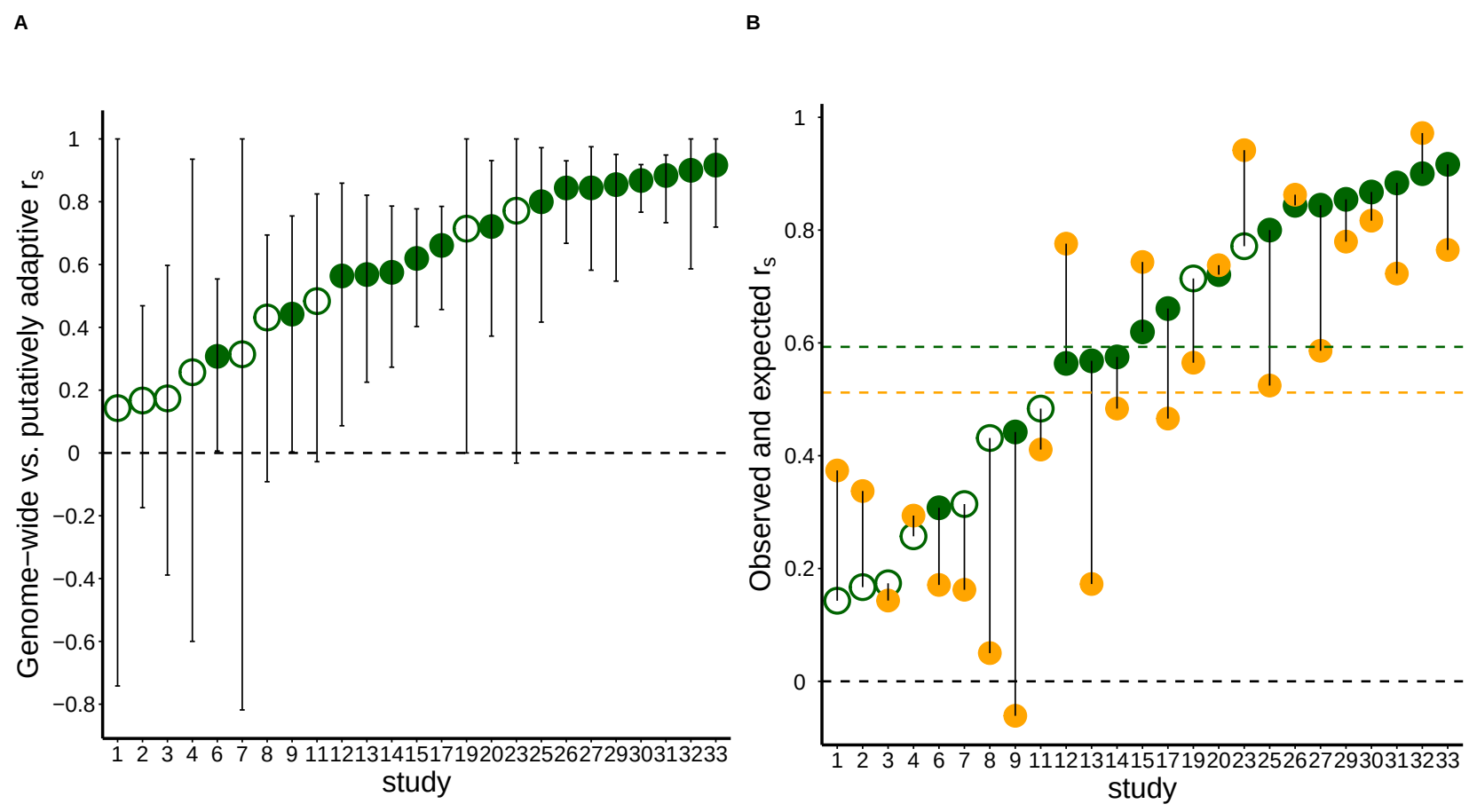

Figure S7: A: The observed Spearman correlation coefficients (green points) and 90% bootstrapped confidence intervals (black lines) of population distinctiveness scores between genome-wide and putatively adaptive SNPs for a subset of studies with $>$1000 SNPs (n = 25 studies). A more positive value indicates a higher prioritization agreement between the two sets of SNPs. The hollow green points represent cases where 90% bootstrapped confidence intervals of the observed correlations overlap zero. B: Observed (green, as in (A)) and expected (orange) Spearman correlations of population distinctiveness scores. The expected correlation for each study compares random subsets (1000 bootstrapped replicates) of genome-wide SNPs with the full genome-wide set. The vertical black lines for each study represent the predictive power of the putatively adaptive SNPs (see text).

We found that putatively adaptive subsets of SNPs show a stronger-than-expected correlation in 16/25 cases. The mean expected Spearman correlation r_s is 0.51 across studies (Figure S7). A two-tailed paired t-test (without removing one outlier study) confirmed this difference ($t(24) = 2.111$, $p = 0.045$) with a mean difference of 0.081 (95% CI: 0.002 to 0.160). These patterns became somewhat weaker (with concomitant loss of power) after removing one additional, outlier study (Benestan et al., 2016) that fell above Q3 + 1.5xIQR or below Q1-1.5xIQR) using the 'identify_outliers' function from the `rstatix` package (Kassambara 2023): $t(23) = 1.784$, $p = 0.088$ with a mean difference of 0.064 (95% CI: -0.010 to 0.137).

Table S3: Impact of six characteristics on the prioritization agreement and the predictive power of putatively adaptive SNPs using only the 25 studies with more than 1000 SNPs. r_s is the Spearman correlation, p is uncorrected p-value, p_c is FDR-corrected p value.

**Study-based characteristics**	**Prioritization agreement**	**Predictive power of putatively adaptive SNPs**
log(study-wide F_{ST})	r_s = 0.12, p = 0.56, p_c = 0.56	r_s = -0.37, p = 0.07, p_c = 0.15
Account for neutral population structure or demography	p = 0.23, p_c = 0.35	p = 0.08, p_c = 0.15
log(number of genome-wide SNPs)	r_s = -0.13, p = 0.52, p_c = 0.56	r_s = -0.25, p = 0.23, p_c = 0.28
log(number of putatively adaptive SNPs)	r_s = 0.54, p = 0.006, p_c = 0.02	r_s = -0.44, p = 0.03, p_c =0.15
log(proportion of putatively adaptive SNPs)	r_s = 0.68, p = 0.0002, p_c = 0.001	r_s = -0.29, p = 0.16, p_c = 0.24
Method Type (F_{ST} vs. non-F_{ST})	p = 0.03, p_c = 0.05	p = 0.34, p_c = 0.34

Study-based characteristics and prioritization agreement: From an ANOVA, it seems that number of putatively adaptive SNPs, proportion of putatively adaptive SNPs, and Method Type (F_{ST} vs. non-F_{ST}) impact each other. A beta-analysis on the impact of putatively adaptive SNPs and proportion of putatively adaptive SNPs on the prioritization agreement shows that log of proportion of putatively adaptive SNPs predicts the prioritization agreement (log of proportion of putatively adaptive SNPs: coefficient = 0.48, p = 0.007, n =25; log of number of putatively adaptive SNPs: coefficient = 0.10, p = 0.51, n = 25).

Study-based characteristics and predictive power of putatively adaptive SNPs: Using 34 studies (all data), we found a negative Spearman correlation between study-wide F_{ST} and predictive power of putatively adaptive SNPs and a negative Spearman correlation between number of putatively adaptive SNPs and predictive power of putatively adaptive SNPs. After repeating our analysis with only the 25 studies that contained more than 1000 genome-wide SNPs, we no longer find a strong Spearman correlation between the predictive power of putatively adaptive SNPs and the two study-based characteristics (see Table S3). With only the 25 studies with more than 1000 genome-wide SNPs, we performed a linear regression and ANOVA to test the impact of log of number of putatively adaptive SNPs and log of study-wide F_{ST} on the predictive power of putatively adaptive SNPs. Without removing one outlier study (Benestan et al., 2016), log of number of putatively adaptive SNPs: estimate = -0.06, p = 0.0066; log of study-wide F_{ST}: estimate = -0.035, p = 0.109 and with removing one outlier study (Benestan et al., 2016), log of number of putatively adaptive SNPs: estimate = -0.055, p = 0.067; log of study-wide F_{ST}: estimate = -0.028, p = 0.25.

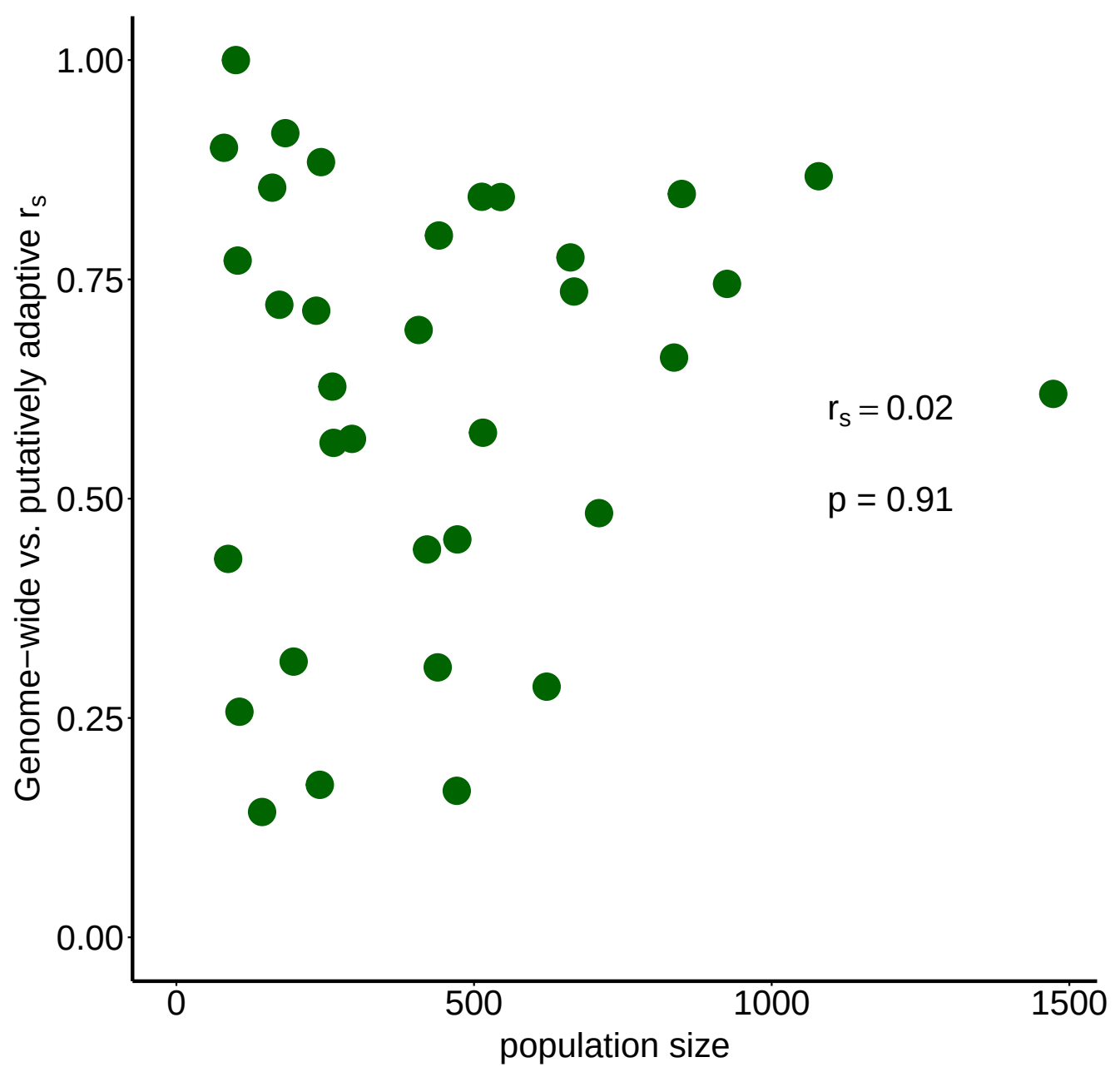

Figure S8: No relationship between number of individuals per population in our final dataset and the correlation between genome-wide and putatively adaptive SNPs. Note that all populations with fewer than 4 individuals were not included in our final dataset in any analysis.

1.3 Details of studies included in the analysis

1. Ruegg et al., 2018 and 2021 generated a dataset of willow flycatcher (*Empidonax traillii*) across its breeding range and wintering locations. After applying 10% missingness cut-off, the dataset consisted of 105000 genome-wide SNPs, 179 putatively adaptive SNPs, 144 individuals, and 6 populations. Data is publicly available on GitHub [https://github.com/eriqande/ruegg-et-al-wifl-genoscapemake-the-genoscape.rmdv] (provided by original authors of the study).

2. Eimanifar et al., 2018 generated a dataset on western honey bees (*Apis mellifera* L.) in the Republic of South Africa. After applying 10% missingness cut-off, the dataset consisted of 2449 genome-wide SNPs, 140 putatively adaptive SNPs, 471 individuals and 29 populations. Raw data is available on Dryad Digital Repository[https://doi.org/10.5061/dryad. 98jh446].

3. White et al., 2013 published a dataset on bank vole (*Myodes glareolus*) in Ireland. After applying 10% missingness cut-off, there were 4340 genome-wide

SNPs, 17 putatively adaptive SNPs, 241 individuals, and 14 populations. Raw data is available on Dryad Digital Repository [https://doi.org/10.5061/dryad.fb782].

4. Schweizer et al., 2016 published a dataset on grey wolf (*Canis lupus*) in Arctic and High Arctic six distinct ecotypes. After applying 10% missingness cut-off, 13092 genome-wide SNPs, 34 putatively adaptive SNPs, 106 individuals, and 6 populations. Raw data is available on Dryad Digital Repository [https://doi.org/10.5061/dryad.8g0s3].

5. Mosca et al., 2016 sampled data on *Pinus mugo* within Italian Alpine regions. After applying 10% missingness cut-off, *Pinus mugo* data consisted of 615 genome-wide SNPs, 89 putatively adaptive SNPs, 622 individuals, and 20 populations. Raw data is published on Dryad Digital Repository [https://doi.org/10.5061/dryad.tm33d]. This Dryad is stores data for Mosca et al., 2012 Mosca et al. 2012 study.

6. Mckown et al., 2014 published data on black cottonwood (*Populus trichocarpa*) from its North American range. After applying 10% missingness cut-off, the dataset consisted of 29,535 genome-wide SNPs, 410 putatively adaptive SNPs, 439 individuals, and 25 populations. Population data came from supplementary files of Geraldes et al., 2014.

7. Royer et al., 2016 produced data on Joshua tree species (*Y.brevifolia*, *Y.jaegeriana*, and hybrids) from Tikaboo Valley, Nevada. After applying 10% missingness cut-off, the dataset consisted of 4603 genome-wide SNPs, 35 putatively adaptive SNPs, 197 individuals and 6 populations.

8. Christmas et al., 2016 generated data on narrow-leaf hopbush (*Dodonaea viscosa* spp. *angustissima*) of Australia. After applying 10% missingness cut-off, the dataset consisted of 7255 genome-wide SNPs, 59 putatively adaptive SNPs, 87 individuals, and 17 populations. The data was obtained via email and supplementary files. Dr. Christmas and Dr. Lowe emailed the population data.

9. Benestan et al., 2016 generated data on American lobster (*Homarus americanus*) sampled across the North American Atlantic Coast. The dataset consisted of 3395 genome-wide SNPs, 7 putatively adaptive SNPs, 421 individuals and 19 populations after applying 10% missingness cut-off. Raw data is

available on Dryad Digital Repository [https://doi.org/10.5061/dryad.5vb8v].

10. Roffler et al., 2016 sampled data on Dall's sheep *Ovis dalli dalli* in Alaska and Yukon Territory available publicly on Dryad Digital Repository [https://doi.org/10.5061/ dryad.kk466]. After applying 10% missingness cut-off, the dataset consisted of 187 genome-wide SNPs, 57 putatively adaptive SNPs, 472 individuals, and 15 populations.

11. Babin et al., 2017 sampled data on the American Eel (*Anguilla rostrata*) from Newfoundland to Florida. The authors emailed me their data and with permission we publish our conversion of their data on Dryad Digital Repository. After applying 10% missingness cut-off, the dataset had 12,098 genome-wide SNPs, 269 putatively adaptive SNPs, 710 individuals, and 13 populations.

12. Guo et al., 2016 published data on Andrew's toad (*Bufo andrewsi*) from Tibetan Plateau's edge on Dryad Digital Repository [https://doi.org/10.5061/dryad.n70c7]. The data consisted of 15,577 genome-wide SNPs, 586 putatively adaptive SNPs, 264 individuals, and 11 populations.

13. De Kort et al., 2014 published data on Black alder (*Alnus glutinosa*) from throughout Europe on Dryad Digital Repository [https://doi.org/10.5061/dryad.rg82f]. After applying 10% missingness cut-off, the dataset consisted of 1714 genome-wide SNPs, 16 putatively adaptive SNPs, 295 individuals and 23 populations.

14. Hurel et al., 2021 sampled data on *Pinus pinaster* in southwestern France. The raw data files are available on Dryad Digital Repository [https://doi.org/10.5061/dryad.r4xgx d2df]. After applying 10% missingness cut-off, the dataset consisted of 6074 genome-wide SNPs, 96 putatively adaptive SNPs, 515 individuals and 33 populations.

15. Depardieu et al., 2021 examined white spruce (*Picea glauca* [Moench] Voss) data across Quebec. The data is available on Dryad Digital Repository [https://doi.org/10.5061/ dryad.6rd6f] and on Github [https://github.com/ClaireDepardieu/Genetic_basis_c The dataset consisted of 6153 raw genome-wide data, 359 putatively adaptive SNPs, 1473 individuals, and 43 populations.

16. Chen et al., 2012 sampled data on Norway spruce trees (*Picea abies*)

in Germany, Russia, Finland, and Sweden. The data is available on Dryad Digital Repository [https://doi.org/10.5061/dryad.82201]. After applying 10% missingness cut-off, the dataset included 375 genome-wide SNPs, 32 putatively adaptive SNPs, 262 individuals, and 18 populations.

17. Xuereb et al., 2022 published data on Coho salmon (*Oncorhynchus kisutch*) in British Columbia on Dryad Digital Repository [https://doi.org/10.5061/dryad.r4xgxd2gx]. I only analyzed Thompson River data for this study. After applying 10% missingness cut-off, the dataset included 9683 genome-wide SNPs, 119 putatively adaptive SNPs, 836 individuals, and 26 populations.

18. Holliday et al., 2010 sampled data on Sitka spruce (*Picea sitchensis*) along the west coast of North America (from Alaska to California). The authors emailed me their data and granted permission to publish 0,1,2,NA (our version of their data) on the Dryad Repository. After removing populations with less than 4 individuals, the dataset incorporated 437 genome-wide SNPs, 35 putatively adaptive SNPs, 407 individuals, and 13 populations.

19. Flanagan et al., 2021 published data on Gulf pipefish (*Syngnathus scovelli*) of southeastern USA. The data is publicly available on Dryad Digital Repository [https://doi.org /10.5061/dryad.12jm63xvh]. After applying 10% missingness cut-off, the dataset contained 2738 genome-wide SNPs, 312 putatively adaptive SNPs, 235 individuals, and 7 populations.

20. Bay et al., 2018 sampled data on yellow warbler (*Setophaga petechia*) in the USA, Canada, Central America, and northern South America. The authors emailed us their data and granted permission to publish the converted version of their data on Dryad. After applying 10% missingness cut-off, the dataset consisted of 104,385 genome-wide SNPs, 1609 putatively adaptive SNPs, 173 individuals, and 19 populations.

21. Chavez-Galarza et al., 2013 sampled data on Iberian honey bee (*Apis mellifera iberiensis*) in the Iberian Peninsula. The data is publicly available on Dryad Digital Repository [https://doi.org/10.5061/dryad.1kk2k] and Dr.Pinto also emailed me data files and granted permission to post our conversion of

their data files on Dryad. After applying 10% missingness cut-off, the dataset contained 434 genome-wide SNPs, 73 putatively adaptive SNPs, 668 individuals, and 23 populations.

22. Keller et al., 2018 expanded on data from Keller et al., 2012 by sampling across balsam poplar's (*Populus balsamifera L*)species range. The dataset is available on Dryad Digital Repository [https://doi.org/10.5061/dryad.gp78p]. After applying 10% missingness cut-off, the dataset contained 279 genome-wide SNPs, 124 putatively adaptive SNPs, 925 individuals, and 83 populations.

23. Funk et al., 2016 published data on island fox (*Urocyon littoralis*) from California Channel Islands on Dryad Digital Repository [https://doi.org/10.5061/dryad.2kn1v]. After applying 10% missingness cut-off, the dataset contained 2,498 genome-wide SNPs, 176 putatively adaptive SNPs, 103 individuals, and 19 populations.

24. Mosca et al., 2016 sampled data on *Pinus cembra* within Italian Alpine regions. After applying 10% missingness cut-off, *Pinus cembra* data consisted of 443 genome-wide SNPs, 85 putatively adaptive SNPs, 662 individuals, and 18 populations. Raw data is published on Dryad Digital Repository [https://doi.org/10.5061/dryad.tm33d]. This Dryad is stores data for Mosca et al., 2012 Mosca et al. 2012 study.

25. Candy et al., 2015 published data on anadromous Pacific smelt (*Thaleichthys pacificus*, Osmeridae) on Dryad Digital Repository [https://doi.org/10.5061/dryad.1797v]. After applying 10% missingness cut-off, the dataset contained 3725 genome-wide SNPs, 157 putatively adaptive SNPs, 441 individuals, and 11 populations.

26. Dallaire et al., 2021 sampled neutral and putatively adaptive data on anadromous Arctic Char (*Salvelinus alpinus*) across Nunavik, southern Baffin Island, and Labrador. Dr. Dallaire emailed me genome-wide SNPs, putatively adaptive SNPs, and population data and granted permission to publish our converted version of their dataset on dryad. After applying 10% missingness cut-off, the dataset comprised of 13,596 genome-wide SNPs, 732 putatively adaptive SNPs, 545 individuals, and 23 populations.

27. Hess et al., 2013 published data on Pacific lamprey (*Entosphenus tridentatus*) on Dryad Digital Repository [https://doi.org/10.5061/dryad.nd853].

After applying 10% missingness cut-off, the dataset comprised of 4,439 genome-wide SNPs, 164 putatively adaptive SNPs, 513 individuals, 21 populations.

28. Milano et al., 2014 published data on European hake populations (*Merluccius merliccius*) sampled from the Atlantic and Mediterranean on Dryad Digital Repository [https://doi.org/10.5061/dryad.7bn22]. After applying 10% missingness cut-off, the dataset comprised of 380 genome-wide SNP data, 30 putatively adaptive SNPs, 849 individuals, 19 populations.

29. Swaegers et al., 2015 collected data on damselfly *Coenagrion scitulum*. The data is available on Dryad [https://doi.org/10.5061/dryad.n0hk7] and Dr. Swaegers also emailed us the data. After applying 10% missingness cut-off, the dataset consisted of 3470 genome-wide SNPs, 566 putatively adaptive SNPs, 161 individuals, and 10 populations.

30. Moore et al., 2014 published data on anadromous Atlantic Salmon (*salmo salar*) covering the entire North American range. The data is available on Dryad [https://doi.org/ 10.5061/dryad.sb601]. After applying 10% missingness cut-off, the dataset included 3192 genome-wide SNPs, 374 putatively adaptive SNPs, 1079 individuals, 50 populations.

31. Mahony et al., 2020 published data on lodgepole pine (*Pinus contorta*) sampled across western Canada on Dryad [https://doi.org/10.5061/dryad.56j8vq8]. I used a subset of 22 populations and a list of putatively adaptive SNPs from Fernandez Fournier et al., 2021 analysis [https://github.com/philippeff/PopulationPrioritization]. After applying 10% missingness cut-off, the dataset included 26431 genome-wide SNPs, 460 putatively adaptive SNPs, 243 individuals, and 22 populations.

32. He et al., 2016 generated data on *Banksia attenuata* (Proteaceae) from southwestern Australia. Dr. He kindly emailed me the datasets and granted permission to publish our converted version of their dataset on Dryad. The dataset included 5701 genome-wide SNPs, 1049 putatively adaptive SNPs, 80 individuals, 9 populations.

33. Li et al., 2021 sampled data on Chimarrichthys fish complex, (*C. davidi*, *C. kishinouyei*, *C. longibarbatus*) found in the Hengduan Mountain Region of

China. They collected fish from Jinsha River, Qingyi River, Dadu River and Yalong River. Dr.Peng emailed me the data and kindly provided permission to publish our converted version of their datasets on Dryad. After applying 10% missingness cut-off, the dataset contained 5834 genome-wide SNPs, 258 putatively adaptive SNPs, 183 individuals, and 9 populations.

34. Cullingham et al., 2014 used data on lodgepole pine (*Pinus contorta* var. *latifolia*) and jack pine (*Pinus banksiana*) and their interspecific hybrids described in Cullingham et al., 2013. Dr. Cullingham kindly emailed me the data and granted permission to publish our converted conversion of their datasets on Dryad. I only used Jackpine data. After applying 10% missingness cut-off, the dataset contained 361 genome-wide SNPs, 25 putatively adaptive SNPs, 100 individuals, and 4 populations. Since two populations had the exact same Shapley Value (during data analysis), I used 3 populations for this. I got the same results using 3 and 4 populations.

References

Ahrens, C. W., Rymer, P. D., Stow, A., Bragg, J., Dillon, S., Umbers, K. D., and Dudaniec, R. Y., 2018. The search for loci under selection: trends, biases and progress. Molecular Ecology 27.6, pp. 1342–1356. DOI: `https://doi.org/10.1111/mec.14549`.

Babin, C., Gagnaire, P.-A., Pavey, S. A., and Bernatchez, L., 2017. RAD-seq reveals patterns of additive polygenic variation caused by spatially-varying selection in the American eel (*Anguilla rostrata*). Genome Biology and Evolution 9.11, pp. 2974–2986. DOI: `https://doi.org/10.1093/gbe/evx226`.

Bay, R. A., Harrigan, R. J., Underwood, V. L., Gibbs, H. L., Smith, T. B., and Ruegg, K., 2018. Genomic signals of selection predict climate-driven population declines in a migratory bird. Science 359.6371, pp. 83–86. DOI: `10.1126/science.aan4380`.

Beaumont, M. A. and Nichols, R. A., 1996. Evaluating loci for use in the genetic analysis of population structure. Proceedings of the Royal Society of London. Series B: Biological Sciences 263.1377, pp. 1619–1626. DOI: https://doi.org/10.1098/rspb.1996.0237.

Benestan, L., Quinn, B. K., Maaroufi, H., Laporte, M., Clark, F. K., Greenwood, S. J., Rochette, R., and Bernatchez, L., 2016. Seascape genomics provides evidence for thermal adaptation and current-mediated population structure in American lobster (*Homarus americanus*). Molecular Ecology 25.20, pp. 5073–5092. DOI: https://doi.org/10.1111/mec.13811.

Bonhomme, M., Chevalet, C., Servin, B., Boitard, S., Abdallah, J., Blott, S., and SanCristobal, M., 2010. Detecting selection in population trees: the Lewontin and Krakauer test extended. Genetics 186.1, pp. 241–262. DOI: 10.1534/genetics.110.117275.

Booker, T. R., 2023. Spatial autocorrelation of the environment influences the patterns and genetics of local adaptation. bioRxiv, pp. 2023–05. DOI: : https://doi.org/10.1101/2023.05.29.542754.

Candy, J. R., Campbell, N. R., Grinnell, M. H., Beacham, T. D., Larson, W. A., and Narum, S. R., 2015. Population differentiation determined from putative neutral and divergent adaptive genetic markers in Eulachon (*Thaleichthys pacificus*, Osmeridae), an anadromous Pacific smelt. Molecular Ecology Resources 15.6, pp. 1421–1434. DOI: https://doi.org/10.1111/1755-0998.12400.

Chávez-Galarza, J., Henriques, D., Johnston, J. S., Azevedo, J. C., Patton, J. C., Muñoz, I., De la Rúa, P., and Pinto, M. A., 2013. Signatures of selection in the Iberian honey bee (*Apis mellifera iberiensis*) revealed by a genome scan analysis of single nucleotide polymorphisms. Molecular Ecology 22.23, pp. 5890–5907. DOI: https://doi.org/10.1111/mec.12537.

Chen, J., Källman, T., Ma, X., Gyllenstrand, N., Zaina, G., Morgante, M., Bousquet, J., Eckert, A., Wegrzyn, J., Neale, D., et al., 2012. Disentangling the roles of history and local selection in shaping clinal variation of allele

frequencies and gene expression in Norway spruce (*Picea abies*). Genetics 191.3, pp. 865–881. DOI: https://doi.org/10.1534/genetics.112.140749.

Christmas, M. J., Biffin, E., Breed, M. F., and Lowe, A. J., 2016. Finding needles in a genomic haystack: targeted capture identifies clear signatures of selection in a nonmodel plant species. Molecular Ecology 25.17, pp. 4216–4233. DOI: https://doi.org/10.1111/mec.13750.

Cribari-Neto, F. and Zeileis, A., 2010. Beta Regression in R. Journal of Statistical Software 34.2, pp. 1–24. DOI: 10.18637/jss.v034.i02.

Cullingham, C. I., Cooke, J. E., and Coltman, D. W., 2013. Effects of introgression on the genetic population structure of two ecologically and economically important conifer species: lodgepole pine (*Pinus contorta* var. *latifolia*) and jack pine (*Pinus banksiana*). Genome 56.10, pp. 577–585. DOI: https://doi.org/10.1139/gen-2013-007.

— 2014. Cross-species outlier detection reveals different evolutionary pressures between sister species. New Phytologist 204.1, pp. 215–229. DOI: https://doi.org/10.1111/nph.12896.

Dallaire, X., Normandeau, É., Mainguy, J., Tremblay, J.-É., Bernatchez, L., and Moore, J.-S., 2021. Genomic data support management of anadromous Arctic Char fisheries in Nunavik by highlighting neutral and putatively adaptive genetic variation. Evolutionary Applications 14.7, pp. 1880–1897. DOI: https://doi.org/10.1111/eva.13248.

De Kort, H., Vandepitte, K., Bruun, H. H., Closset-Kopp, D., Honnay, O., and Mergeay, J., 2014. Landscape genomics and a common garden trial reveal adaptive differentiation to temperature across Europe in the tree species *Alnus glutinosa*. Molecular Ecology 23.19, pp. 4709–4721. DOI: https://doi.org/10.1111/mec.12813.

Depardieu, C., Gérardi, S., Nadeau, S., Parent, G. J., Mackay, J., Lenz, P., Lamothe, M., Girardin, M. P., Bousquet, J., and Isabel, N., 2021. Connecting tree-ring phenotypes, genetic associations and transcriptomics to deci-

pher the genomic architecture of drought adaptation in a widespread conifer. Molecular Ecology 30.16, pp. 3898–3917. DOI: https://doi.org/10.1111/mec.15846.

Eimanifar, A., Brooks, S. A., Bustamante, T., and Ellis, J. D., 2018. Population genomics and morphometric assignment of western honey bees (*Apis mellifera L.*) in the Republic of South Africa. BMC Genomics 19, pp. 1–26. DOI: https://doi.org/10.1186/s12864-018-4998-x.

Excoffier, L., Hofer, T., and Foll, M., 2009. Detecting loci under selection in a hierarchically structured population. Heredity 103.4, pp. 285–298. DOI: https://doi.org/10.1038/hdy.2009.74.

Fernandez-Fournier, P., Lewthwaite, J. M., and Mooers, A. Ø., 2021. Do we need to identify adaptive genetic variation when prioritizing populations for conservation? Conservation Genetics 22, pp. 205–216. DOI: https://doi.org/10.1007/s10592-020-01327-w.

Flanagan, S. P., Rose, E., and Jones, A. G., 2021. The population genomics of repeated freshwater colonizations by Gulf pipefish. Molecular Ecology 30.7, pp. 1672–1687. DOI: https://doi.org/10.1111/mec.15841.

Funk, W. C., Lovich, R. E., Hohenlohe, P. A., Hofman, C. A., Morrison, S. A., Sillett, T. S., Ghalambor, C. K., Maldonado, J. E., Rick, T. C., Day, M. D., et al., 2016. Adaptive divergence despite strong genetic drift: genomic analysis of the evolutionary mechanisms causing genetic differentiation in the island fox (*Urocyon littoralis*). Molecular Ecology 25.10, pp. 2176–2194. DOI: https://doi.org/10.1111/mec.13605.

Geraldes, A., Farzaneh, N., Grassa, C. J., McKown, A. D., Guy, R. D., Mansfield, S. D., Douglas, C. J., and Cronk, Q. C., 2014. Landscape genomics of *Populus trichocarpa*: the role of hybridization, limited gene flow, and natural selection in shaping patterns of population structure. Evolution 68.11, pp. 3260–3280. DOI: https://doi.org/10.1111/evo.12497.

Günther, T. and Coop, G., 2013. Robust identification of local adaptation from allele frequencies. Genetics 195.1, pp. 205–220. DOI: https://doi.org/10.1534/genetics.113.152462.

Guo, B., Lu, D., Liao, W. B., and Merilä, J., 2016. Genomewide scan for adaptive differentiation along altitudinal gradient in the Andrew's toad *Bufo andrewsi*. Molecular Ecology 25.16, pp. 3884–3900. DOI: https://doi.org/10.1111/mec.13722.

He, T., D'Agui, H., Lim, S. L., Enright, N. J., and Luo, Y., 2016. Evolutionary potential and adaptation of *Banksia attenuata* (Proteaceae) to climate and fire regime in southwestern Australia, a global biodiversity hotspot. Scientific Reports 6.1, p. 26315. DOI: https://doi.org/10.1038/srep26315.

Hess, J. E., Campbell, N. R., Close, D. A., Docker, M. F., and Narum, S. R., 2013. Population genomics of Pacific lamprey: adaptive variation in a highly dispersive species. Molecular Ecology 22.11, pp. 2898–2916. DOI: https://doi.org/10.1111/mec.12150.

Hoban, S., Kelley, J. L., Lotterhos, K. E., Antolin, M. F., Bradburd, G., Lowry, D. B., Poss, M. L., Reed, L. K., Storfer, A., and Whitlock, M. C., 2016. Finding the genomic basis of local adaptation: pitfalls, practical solutions, and future directions. The American Naturalist 188.4, pp. 379–397. DOI: https://doi.org/10.1086/688018.

Holliday, J. A., Ritland, K., and Aitken, S. N., 2010. Widespread, ecologically relevant genetic markers developed from association mapping of climate-related traits in Sitka spruce (*Picea sitchensis*). New Phytologist 188.2, pp. 501–514. DOI: https://doi.org/10.1111/j.1469-8137.2010.03380.x.

Hurel, A., Miguel, M. de, Dutech, C., Desprez-Loustau, M.-L., Plomion, C., Rodríguez-Quilón, I., Cyrille, A., Guzman, T., Alía, R., González-Martínez, S. C., et al., 2021. Genetic basis of growth, spring phenology, and susceptibility to biotic stressors in maritime pine. Evolutionary Applications 14.12, pp. 2750–2772. DOI: https://doi.org/10.1111/eva.13309.

Kassambara, A., Feb. 2023. Rstatix: Pipe-friendly framework for basic statistical tests. Package 'rstatix'. DOI: 10.32614/cran.package.rstatix. URL: https://CRAN.R-project.org/package=rstatix.

Keller, S. R., Chhatre, V. E., and Fitzpatrick, M. C., 2018. Influence of range position on locally adaptive gene–environment associations in Populus flowering time genes. Journal of Heredity 109.1, pp. 47–58. DOI: https://doi.org/10.1093/jhered/esx098.

Keller, S. R., Levsen, N., Olson, M. S., and Tiffin, P., 2012. Local adaptation in the flowering-time gene network of balsam poplar, *Populus balsamifera L.* Molecular Biology and Evolution 29.10, pp. 3143–3152. DOI: https://doi.org/10.1093/molbev/mss121.

Li, Y., Burridge, C. P., Lv, Y., and Peng, Z., 2021. Morphometric and population genomic evidence for species divergence in the *Chimarrichthys* fish complex of the Tibetan Plateau. Molecular Phylogenetics and Evolution 159, p. 107117. DOI: https://doi.org/10.1016/j.ympev.2021.107117.

Lotterhos, K. E. and Whitlock, M. C., 2014. Evaluation of demographic history and neutral parameterization on the performance of F_{ST} outlier tests. Molecular Ecology 23.9, pp. 2178–2192. DOI: https://doi.org/10.1111/mec.12725.

Mahony, C. R., MacLachlan, I. R., Lind, B. M., Yoder, J. B., Wang, T., and Aitken, S. N., 2020. Evaluating genomic data for management of local adaptation in a changing climate: A lodgepole pine case study. Evolutionary Applications 13.1, pp. 116–131. DOI: https://doi.org/10.1111/eva.12871.

McKown, A. D., Klápště, J., Guy, R. D., Geraldes, A., Porth, I., Hannemann, J., Friedmann, M., Muchero, W., Tuskan, G. A., Ehlting, J., et al., 2014. Genome-wide association implicates numerous genes underlying ecological trait variation in natural populations of *Populus trichocarpa*. New Phytologist 203.2, pp. 535–553. DOI: https://doi.org/10.1111/nph.12815.

Milano, I., Babbucci, M., Cariani, A., Atanassova, M., Bekkevold, D., Carvalho, G. R., Espiñeira, M., Fiorentino, F., Garofalo, G., Geffen, A. J., et al., 2014.

Outlier SNP markers reveal fine-scale genetic structuring across European hake populations (*Merluccius merluccius*). Molecular Ecology 23.1, pp. 118–135. DOI: https://doi.org/10.1111/mec.12568.

Moore, J.-S., Bourret, V., Dionne, M., Bradbury, I., O'Reilly, P., Kent, M., Chaput, G., and Bernatchez, L., 2014. Conservation genomics of anadromous Atlantic salmon across its North American range: outlier loci identify the same patterns of population structure as neutral loci. Molecular Ecology 23.23, pp. 5680–5697.

Mosca, E., Eckert, A., Di Pierro, E., Rocchini, D., La Porta, N., Belletti, P., and Neale, D., 2012. The geographical and environmental determinants of genetic diversity for four alpine conifers of the European Alps. Molecular Ecology 21.22, pp. 5530–5545.

Mosca, E., Gugerli, F., Eckert, A. J., and Neale, D. B., 2016. Signatures of natural selection on *Pinus cembra* and *P. mugo* along elevational gradients in the Alps. Tree Genetics & Genomes 12, pp. 1–15. DOI: https://doi.org/10.1007/s11295-015-0964-9.

Roffler, G. H., Amish, S. J., Smith, S., Cosart, T., Kardos, M., Schwartz, M. K., and Luikart, G., 2016. SNP discovery in candidate adaptive genes using exon capture in a free-ranging alpine ungulate. Molecular Ecology Resources 16.5, pp. 1147–1164. DOI: 10.1111/1755-0998.12560.

Royer, A. M., Streisfeld, M. A., and Smith, C. I., 2016. Population genomics of divergence within an obligate pollination mutualism: Selection maintains differences between Joshua tree species. American Journal of Botany 103.10, pp. 1730–1741. DOI: https://doi.org/10.3732/ajb.1600069.

Ruegg, K., Anderson, E. C., Somveille, M., Bay, R. A., Whitfield, M., Paxton, E. H., and Smith, T. B., 2021. Linking climate niches across seasons to assess population vulnerability in a migratory bird. Global Change Biology 27.15, pp. 3519–3531. DOI: https://doi.org/10.1111/gcb.15639.

Ruegg, K., Bay, R. A., Anderson, E. C., Saracco, J. F., Harrigan, R. J., Whitfield, M., Paxton, E. H., and Smith, T. B., 2018. Ecological genomics predicts

climate vulnerability in an endangered southwestern songbird. Ecology Letters 21.7, pp. 1085–1096. DOI: https://doi.org/10.1111/ele.12977.

Savolainen, O., Pyhäjärvi, T., and Knürr, T., 2007. Gene flow and local adaptation in trees. Annual Review of Ecology, Evolution, and Systematics 38.1, pp. 595–619.

Schweizer, R. M., Robinson, J., Harrigan, R., Silva, P., Galverni, M., Musiani, M., Green, R. E., Novembre, J., and Wayne, R. K., 2016. Targeted capture and resequencing of 1040 genes reveal environmentally driven functional variation in grey wolves. Molecular Ecology 25.1, pp. 357–379. DOI: https://doi.org/10.1111/mec.13467.

Sork, V., Aitken, S., Dyer, R., Eckert, A., Legendre, P., and Neale, D., 2013. Putting the landscape into the genomics of trees: approaches for understanding local adaptation and population responses to changing climate. Tree Genetics & Genomes 9, pp. 901–911. DOI: https://doi.org/10.1007/s11295-013-0596-x.

Swaegers, J., Mergeay, J., Van Geystelen, A., Therry, L., Larmuseau, M., and Stoks, R., 2015. Neutral and adaptive genomic signatures of rapid poleward range expansion. Molecular Ecology 24.24, pp. 6163–6176. DOI: https://doi.org/10.1111/mec.13462.

White, T. A., Perkins, S. E., Heckel, G., and Searle, J. B., 2013. Adaptive evolution during an ongoing range expansion: the invasive bank vole (*Myodes glareolus*) in Ireland. Molecular Ecology 22.11, pp. 2971–2985. DOI: https://doi.org/10.1111/mec.12343.

Xuereb, A., Rougemont, Q., Dallaire, X., Moore, J.-S., Normandeau, E., Bougas, B., Perreault-Payette, A., Koop, B. F., Withler, R., Beacham, T., et al., 2022. Re-evaluating Coho salmon (*Oncorhynchus kisutch*) conservation units in Canada using genomic data. Evolutionary Applications 15.11, pp. 1925–1944. DOI: https://doi.org/10.1111/eva.13489.
